# Enabling direct microcalorimetric measurement of metabolic activity and exothermic reactions onto microfluidic platforms via heat flux sensor integration

**DOI:** 10.1038/s41378-023-00525-z

**Published:** 2023-05-09

**Authors:** Signe L. K. Vehusheia, Cosmin Roman, Olivier Braissant, Markus Arnoldini, Christofer Hierold

**Affiliations:** 1 Micro and Nanosystems, Department of Mechanical and Process Engineering, ETH Zurich, Tannenstrasse 3, 8092 Zurich, Switzerland; 2 Center of Biomechanics and Biocalorimetry, Department of Biomedical Engineering, University of Basel, Hegenheimermattweg 167C, 4123 Allschwil, Switzerland; 3 Laboratory for Food Immunology, Department of Health Sciences and Technology, ETH Zurich, Otto-Stern-Weg 3, 8093 Zurich, Switzerland

**Keywords:** Microfluidics, Sensors, Biosensors

## Abstract

All biological processes use or produce heat. Traditional microcalorimeters have been utilized to study the metabolic heat output of living organisms and heat production of exothermic chemical processes. Current advances in microfabrication have made possible the miniaturization of commercial microcalorimeters, resulting in a few studies on the metabolic activity of cells at the microscale in microfluidic chips. Here we present a new, versatile, and robust microcalorimetric differential design based on the integration of heat flux sensors on top of microfluidic channels. We show the design, modeling, calibration, and experimental verification of this system by utilizing *Escherichia coli* growth and the exothermic base catalyzed hydrolysis of methyl paraben as use cases. The system consists of a Polydimethylsiloxane based flow-through microfluidic chip with two 46 µl chambers and two integrated heat flux sensors. The differential compensation of thermal power measurements allows for the measurement of bacterial growth with a limit of detection of 1707 W/m^3^, corresponding to 0.021OD (2 ∙ 10^7^ bacteria). We also extracted the thermal power of a single *Escherichia coli* of between 1.3 and 4.5 pW, comparable to values measured by industrial microcalorimeters. Our system opens the possibility for expanding already existing microfluidic systems, such as drug testing lab-on-chip platforms, with measurements of metabolic changes of cell populations in form of heat output, without modifying the analyte and minimal interference with the microfluidic channel itself.

## Introduction

General investigations of bacterial and cellular systems on a micro scale are of high interest for immunology^1^, diagnostics^2,3^, and pharmacology^4^. Experimental systems have been developed to mimic tumors^5^, or organs such as the lung^6^ or kidney^7^, and the drug efficiency against bacteria^8^ or of cancer biopsies^9^ to study their behavior and responses. Current approaches also allow for smaller sample size and mimicking of in vivo systems in in vitro environments in the laboratory. All of these investigations focus on early detection of behavioral changes using different approaches such as optical inspection with fluorescent stains or different biochemical markers. Changes in the metabolic behavior of biological organisms could indicate disturbances in the organismal homeostasis, or responses to successful drug delivery.

In most biological processes heat is produced as a byproduct, which heats up the system and its environment^10–12^, yielding important additional information to optical inspections and biochemical markers. The heat produced can be observed on the macroscale as body heat or quantified on the microscale through the exothermic chemical reactions of the metabolism of cells. Glucose respiration, glucose fermentation, and lactose fermentation, are examples of metabolic processes which dominate the heat output when measuring bacterial growth in microcalorimeters, making it possible to correlate the heat produced on the available oxygen and glucose in the system^13^. Different bacterial strains show different metabolic activity in the same environment^14^, which could be used for bacterial strain characterization from the heat measured. Any changes in organism behavior are accompanied by changes in metabolic processes and can thus be related back to changes in the heat output.

Microcalorimetry was developed to measure the metabolic heat output of small samples of cells and chemical reactions. It has been applied in the fields of microbiology^13–15^, soil science^16^, food science^17^, and medicine^18^, as the metabolic change of cells can be measured through heat flow from the sample^13^. The extracted thermograms from the microcalorimetric data reflect the chemical conversion of energy by each individual cell^10^, which can be described with Hess’s law^19^. Microcalorimetry has been used to detect infections or contaminations of samples in medicine^18^ and food sciences^17^, respectively, showing that such detrimental processes can indeed be identified using their heat signatures. However, traditional microcalorimeters are expensive, large and bulky machines, and are hardly compatible with other characterization methods (e.g., optical density for microbial cultures). In order to overcome these, microcalorimetric chips^20,21^, nanocalorimeters^12,22^, and picocalorimeters^23^ have been developed. These systems have allowed important scientific progress such as single cell thermal power measurements^20,23^. Further research has been conducted on different biologically relevant exothermic chemical reactions in microcalorimetric chips^22–25^, both in open-type chips^26–29^, which are limited to experiments of a few minutes, and closed-type chips. Lerchner et al. measured the changes in heat output of the response of a bacterial biofilm to activation and deactivation using a calorimetric chip^30,31^. They also measured the heat output of droplets of bacteria in flow through chip microcalorimeters^32^. Current micro- and nanocalorimetric chips are designed as essentially miniaturized versions of instrumentation microcalorimeters. Their central focus being on measurement accuracy, they often include additional parts such as heaters, thermally insulating layers and PID controllers to ensure the thermal stability of the system^33,34^. The focus on accuracy comes at the expense of design flexibility of the sample chamber. Consequently, micro- and nanocalorimetric chips would be difficult to adapt to measuring metabolic heat changes in complex microfluidic systems such as lab-on-chip or organ-on-chip.

Here, we present a new approach to measuring biological thermal power output. Instead of bringing the sample to the microcalorimetric chip, we propose integrating heat flux sensors onto pre-existent microfluidic platforms through an easy add-on fabrication process. Leveraging the nature of heat flux measurements, we can directly measure the thermal power produced by a sample. To reduce the influence of environmental thermal fluctuations and thereby relaxing the requirements of thermal stability we introduce a differentially compensating approach. The two channels are calibrated thermally based on the exothermic chemical reaction of the base catalyzed hydrolysis of methyl paraben. We detect metabolic heat of bacteria during the exponential growth phase and estimate the thermal power produced by a single bacterium, which agrees well with previous measurements in literature.

In the following sections we will first report the thermal calibration of the microfluidic chip using the exothermic base catalyzed hydrolysis of methyl paraben. With the chemical calibration we determined a heat transfer fraction describing the heat measured by the sensor in relation to the total produced heat in the channel. We were able to relate the heat transfer fraction to a modeled heat transfer fraction and analyze the influence of the flow environment on the heat transfer fraction. Following the calibration of the microfluidic chip, we measured the bacterial metabolic heat output in the microfluidic system in correlation with the optically measured bacterial growth. From this data we were able to extract the thermal power produced by a single bacterium, and the limit of detection for bacteria measured with the system.

## Experimental results and discussion

### Experimental setup and operation

**Fig. 1:**
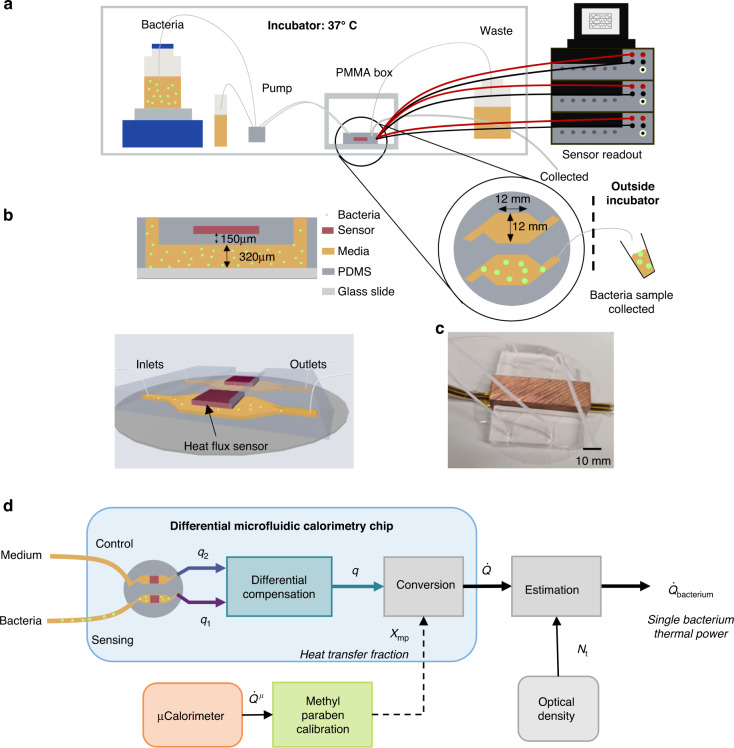
Experimental setup of the microfluidic calorimetry chip and the overview of experiments performed to determine the thermal power of a single bacterium. **a** 37 °C incubator chamber enclosing a bacteria container on top of a magnetic stirrer, reference media, and a peristaltic pump. **b** Schematic side and 3D-tilted views of the microfluidic chip with one channel utilized for sensing and the other for common-mode rejection. **c** Picture of the microfluidic chip with a copper block (for temperature stabilization) covering the heat flux sensors. **d** Overview over the different experiments in the microfluidic chip (calibration and bacterial measurement). Thermal calibration was performed using an exothermic methyl paraben. The heat flux bacterial growth was measured by applying a differential compensation method to the data coming from the two sensors, labeled sensing and control. The measured optical density of bacteria allows estimating the average thermal power of a single bacterium

Figure 1a shows the experimental setup developed for the purpose of measuring the thermal activity of bacteria. At the core of this setup is a differential microfluidic calorimetry chip with a pair of integrated heat flux sensors (gSKIN^®^ XP 26C). The microfluidic chip, sketched in Fig. 1b and shown in Fig. 1c, is fabricated using Polydimethylsiloxane (PDMS) and fused to a glass slide. The process of fabrication, as described in detail in the methods section, uses common microfluidic processing for the microfluidic channels with an additional PDMS encapsulation step of the heat flux sensors prior to PDMS fusing with the glass slide. Both microfluidic channels were fabricated identically (width = 12 mm, length = 12 mm) with 320 µm height over which a heat flux sensor was placed with a 150 µm thick layer of PDMS in between. The heat flux sensors are placed in proximity to the channels in order to maximize the heat transfer fraction through the sensors. Similar to the principle of a flow microcalorimeter^35^, the sample fluid was flown using a peristaltic pump through both microfluidic channels while measuring the heat flux with the two sensors. The two heat flux sensors enable differential compensation of the measured signal, which, as shown later, significantly improves the signal to noise ratio by reducing extrinsic common-mode fluctuations (e.g., local temperature, flow rate in the two sensing channels. In addition, a piece of copper was placed above the heat flux sensors for stabilization of environmental thermal fluctuations (Fig. 1c). An overview over the key performance metrics including the limit of detection, sensor resolution, time constant, and thermal conductivity can be found in Table S1 in the Supplementary Information (SI).

The schematic of the three different experimental setups is described in Fig. 1d, namely as applied for a bacterial experiment in 37 °C incubator, a methyl paraben calibration experiment in an oven, and a microcalorimetry experiment. Firstly, the heat transfer efficiency of the microfluidic chip is determined through a thermal calibration experiment using the base catalyzed exothermic reaction of methyl paraben and Sodium hydroxide (NaOH), as described in the SI in Fig. S1. From the calibration experiment we determine a heat transfer fraction, *χ*_mp_, which relates the exothermic heat input in the channel to the heat measured by the heat flux sensor. The total heat produced by the exothermic chemical reaction per unit volume is determined by a commercial microcalorimeter. In the bacterial experiment, we extract a heat signal, *q*, of the bacterial growth by applying a differential compensation method on the sensing (lysogeny broth (LB) and bacteria) and control/compensation (LB) heat flux data. Subsequently, the heat signal, *q*, of the microfluidic calorimetry chip is converted into the total thermal power of the bacteria in the microfluidic chip channel, $\dot Q$, via the area of the sensor *A*_sensor_ and the heat transfer fraction, *χ*_mp_. The optical density (OD) measurement of bacteria, collected using a piece of tubing from the outlet of the microfluidic channel fed through to the outside of the incubator in parallel to the bacterial heat flux measurement allows the determination of total bacterial population, *N*_t_, as a function of time (as described in the SI in Fig. S2), which leads to the estimation of the thermal power of a single bacterium (or heat rate) $\dot Q_{{{{\mathrm{bacteria}}}}}$.

### Methyl paraben heat transfer fraction in microfluidic chip

**Fig. 2:**
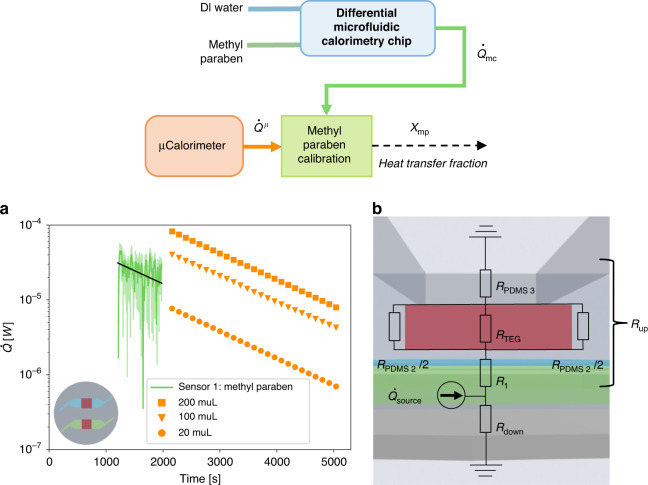
Determination of the heat transfer fraction χmp. **a** Calibration experiments with methyl paraben. Comparative plot between the heat rate at the output of the differential microfluidic calorimetry chip $\dot Q_{{{{\mathrm{mc}}}}}$ (raw data are shown with a lighter green color line and a 10 point moving-average with a darker green color line) and microcalorimetric ampoules $\dot Q^{{{\mu }}/{{{\mathrm{V}}}}}$ (20 μl, 100 μl, and 200 μl shown in orange and discussed in Section 5 in the SI). The increase in heat upon the exothermic reaction of methyl paraben $\dot Q_{{{{\mathrm{mc}}}}}$ was determined the same way as upon addition of bacteria as discussed in the next section (for detailed discussion see Section 5 in the SI). The time origin is at the time where methyl paraben was mixed with NaOH for both experiments. **b** A lumped element model of the microfluidic chip to determine the heat transfer fraction. Each thermal resistance represents either a PDMS, glass, Poly(methyl methacrylate) PMMA or air layer of a certain geometry, or multiple layers and interfaces. $R_1 = R_{{{{\mathrm{conv}}}}} + R_{{\rm{PDMS1}}}$ and $R_{{{{\mathrm{down}}}}} = R_{{{{\mathrm{conv}}}}} + R_{{{{\mathrm{glass}}}}} + R_{{{{\mathrm{air}}}}} + R_{{{{\mathrm{PMMA}}}}}$, with *R*_conv_ as the convective heat transport between the channel and the PDMS

Our thermal calibration experiment was set up according to the description by O’Neill et al.^36^ using the first order reaction of NaOH catalyzed hydrolysis of methyl paraben. We performed a microcalorimetric experiment (commercial microcalorimeter TAMII nano (Waters/TA, Delaware, USA)) for three different volumes, 20, 100, and 200 μl. The ratio between the volumes was the same as the ratio between the heat measured by these volumes at each time point considered. The microcalorimetric experiment served as the reference heat per volume and the slope of heat decrease *k*. The thermal power measured in the microfluidic calorimetric chip (46 μl) was compared to the reference (as shown in Fig. 2a), to determine a heat transfer fraction *χ*_mp_. A lumped element model (LEM) of the microfluidic system was developed, as shown in Fig. 2b, to theoretically determine a heat transfer fraction comparable to the experimentally determined heat transfer fraction *χ*_mp_.

### Experimental determination of heat transfer fraction

The heat transfer fraction of each channel was determined by extrapolating the data toward the *y*-axis intercept (Fig. 2a), which is the time at which 0.5 M NaOH was mixed with methyl paraben. The data were fitted with the following general function:

1 $$\ln (\dot Q) = \ln (\dot Q_0) + k \cdot t$$

With $\dot Q$ as the measured heat flux, representing either $\dot Q_{{{{\mathrm{mc}}}}}$ (microfluidic calorimetric chip) or $\dot Q^{\upmu}$ (commercial microcalorimetric, $\dot Q^{{{\mu }}/{{{\mathrm{V}}}}}$-not volumetrically normed), and $\dot Q_0$, representing either $\dot Q_{{{{\mathrm{mc}}}}0}$ or $\dot Q_0^{\upmu}$, as the heat flux at the *y*-axis intercept, *k* as the slope of the heat increase, and *t* as the time. We fit the heat decrease rate, *k*, determined from the microcalorimetric data to our data measured from the microfluidic chip. We then extracted the *y*-axis intercept, $\dot Q_{{{{\mathrm{mc}}}}0}$, for the channel to determine the heat flow measured in our system at 46 μl. The *y*-axis intercept of the microfluidic microcalorimeter was compared and the volumetrically normed ratios $\dot Q^{\mu} = \dot Q^{{{\mu }}/{{{\mathrm{V}}}}}V_{{{{\mathrm{mc}}}}}/V^{\mu}$ were compared between the systems, yielding $\chi _{{{{\mathrm{mp}}}}} = \dot Q_{{{{\mathrm{m}}}}0}/\dot Q_0^{\upmu}$ being 77% ± 8% for methyl paraben in channel 1 (error propagation to be found in Section 6 in the SI).

### Comparison between modeled and measured heat transfer fraction

The heat transfer fraction *χ*_mp_ was estimated by the lumped element model sketched in Fig. 2b. The thermal resistance of conduction and convection were expressed as $R_{{{{\mathrm{conduction}}}}} = l/(k_{{{{\mathrm{material}}}}}A_{{{{\mathrm{surface}}}}})$ and $R_{{{{\mathrm{convection}}}}} = 1/(h_{{{{\mathrm{convection}}}}}A_{{{{\mathrm{surface}}}}})$, respectively. The heat transfer fraction between heat produced in the channel and the heat measured by the heat flux sensor was expressed as:

2 $$\chi _{{{{\mathrm{mp}}}}} = \frac{{\dot Q_{{{{\mathrm{TEG}}}}}}}{{\dot Q_{{{{\mathrm{source}}}}}}} \cdot 100{{{\mathrm{\% }}}} = \frac{{R_{{{{\mathrm{down}}}}}}}{{R_{{{{\mathrm{down}}}}} + R_{{{{\mathrm{up}}}}}}}\frac{{R_{{{{\mathrm{PDMS}}}}2}}}{{R_{{{{\mathrm{PDMS}}}}2} + R_{{{{\mathrm{TEG}}}}}}} \cdot 100{{{\mathrm{\% }}}}$$

With $\dot Q_{{{{\mathrm{TEG}}}}}$ as the heat going through the sensor, $\dot Q_{{{{\mathrm{source}}}}}$ as the total heat produced within the channel, $R_{{{{\mathrm{down}}}}} = R_{{{{\mathrm{conv}}}}} + R_{{{{\mathrm{glass}}}}} + R_{{{{\mathrm{air}}}}} + R_{{{{\mathrm{PMMA}}}}}$ as the downwards material layers in series (glass slide, air and PMMA stand), *R*_up_, as the layers of material as illustrated in Fig. 2b, *R*_PDMS2_ as the layer of PDMS on each sides of the sensor, and *R*_TEG_ as the sensor itself. In the LEM, we consider the thermal pathways in the vertical directions neglecting the horizontal directions as illustrated in Fig. 2b. Furthermore, because the small time constants of the microfluidic chip are much smaller than rate of change in thermal power observed in both the calibration and the biological experiments (as the same microfluidic chip is used in both experiments), we disregard the transience of the system. A copper piece was placed on top of the PDMS, *R*_PDMS3_, which has a significant smaller thermal resistance than PDMS, was therefore not considered for the LEM as we consider it to have the same temperature as the ambient. A detailed explanation of the parameters can be found in Section 7 in the SI. For all calculations, we considered the relevant volume to be the 12 × 12 mm^2^ surface underneath the 10 × 10 mm^2^ surface of the sensor. With the mentioned assumptions of the LEM model, the heat transfer fraction estimated was equivalent to the experimentally determined heat transfer fraction of the microfluidic calorimeter chip methyl paraben experiment. The estimated value of *χ*_mp_ *of* 73% is within the experimentally extracted heat transfer fraction of 77% ± 8%, as shown in Section 7 of the SI.

### Analysis of the influence of the flow environment

Through investigations of the flow regime of the microfluidic chip, we determined that the heat transfer coefficient of our system is independent of the flow rate for the flow regime we use (see Section 8 in the SI for in depth discussion). In order to evaluate this, we studied the heat transfer environment in the microfluidic calorimetric chip under flow. In the methyl paraben experiment the flow rate in the channel was 98.75 μl/min, whereas in the *Escherichia coli* (*E. coli)* experiment it was 11.25 μl/min. The thermal time constant of our microfluidic calorimetric chip is smaller than that of common microcalorimeters, mainly due to the large difference in mass. This becomes clear when calculating the time constant of each individual material layer, $\tau \le 9\;s$. Furthermore, the heat transfer coefficient calculated for our system was determined to be in a regime which is flow rate independent and only dependent on the geometry of the channel as suggested in literature^37^ (further elaborated in Section 9 in the SI). This is in line with published measurements^36,38^ showing that the heat flow had at most a small influence on the thermal volume, however it was found that different systems showed either no dependence or a slight dependence. We, therefore, considered the heat transfer environment of our microfluidic calorimetric chip to be flow rate independent.

### Measurement of heat produced by E. coli MG1655

To determine the heat produced by bacterial growth two heat flux sensors were used; one above the channel containing LB and bacteria for the heat measurement and the other channel containing LB for the common-mode rejection. Applying a differential compensation method described in detail below, the total heat measured by the bacterial growth is determined. We determine the single bacterium thermal power in the exponential phase, and the limit of detection of the microfluidic chip platform.

**Fig. 3:**
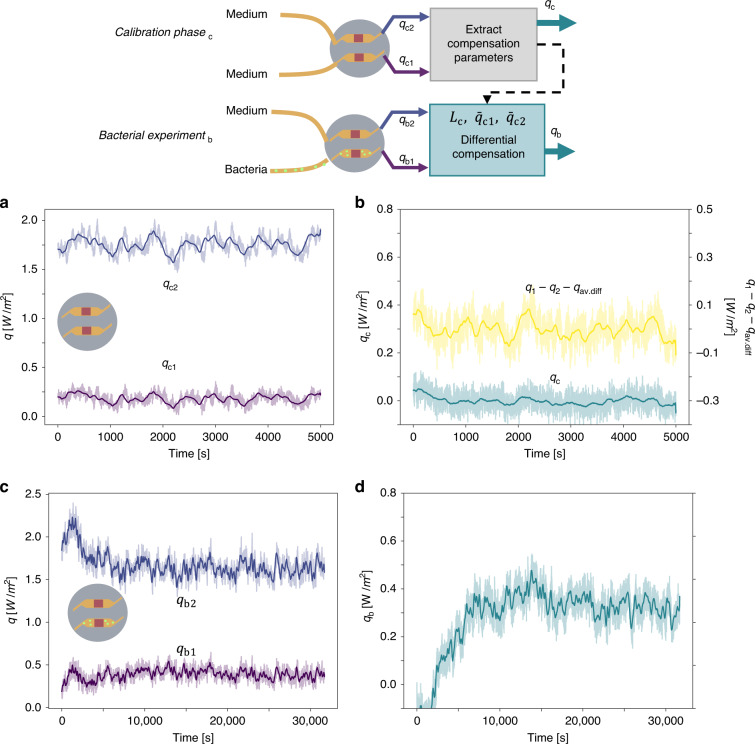
Heat flux measurements and differential compensation of bacterial thermal activity. **a**, **b** Calibration phase (q_c_), where both sensors contain LB medium only and **c**, **d** Bacterial experiment (q_b_), where the sensing channel contains *E. coli* bacteria in LB medium and the compensation channel LB medium only. In (**a**)–(**d**) raw data are shown with a lighter color line and a 200-point moving-average with a darker color line. Measured heat fluxes *q*_1_**–** and *q*_2_**–** in the calibration phase (**a**) and bacterial experiment (**c**), respectively. **b** Simple heat flux difference $q_1 - q_2 - q_{{{{\mathrm{avg}}}}.{{{\mathrm{diff}}}}}$ (yellow line, right axis) and differentially compensated heat flux *q*_c_ (cyan line, left axis), in the calibration phase. **d** Differentially compensated heat flux *q*_b_ upon addition of bacteria (**b**) and bacterial experiment (**d**), respectively. Details can be found in Section 10 in the SI

Figure 3a shows the output of the two heat flux sensors when both channels are being filled with LB medium only. Each channel displays a baseline offset, namely $\bar q_{{{{\mathrm{c}}}}1}$ for the sensing and $\bar q_{{{{\mathrm{c}}}}2}$ for the control channel (here overbar denotes time-average). While the origin of the offset may be relevant for future improvements of the system, in this work, we have devised a differential compensation scheme to cancel out these offsets and other common-mode fluctuations. Figure 3b (right axis) shows that a simple difference between the two offset-canceled channels, namely $q_{{{{\mathrm{c}}}}1} - q_{{{{\mathrm{c}}}}2} - \left( {\bar q_{{{{\mathrm{c}}}}1} - \bar q_{{{{\mathrm{c}}}}2}} \right) = q_{{{{\mathrm{c}}}}1} - q_{{{{\mathrm{c}}}}2} - q_{{{{\mathrm{av}}}}.{{{\mathrm{diff}}}}}$ does result, as expected, in a differential signal with zero offset. However, a comparison of Fig. 3a, b reveals that this differential signal, does not reduce the correlation visible in both heat flux signals in Fig. 3a.

Based on this observation, we propose an improved differential compensation method. In essence, the heat signal is still computed as a difference $q = q_1 - q_2^ \ast$, but a linearly corrected signal $q_2^ \ast = L_{{{\mathrm{c}}}} \cdot \left( {q_2 - \bar q_{{{{\mathrm{c}}}}2}} \right) + \bar q_{{{{\mathrm{c}}}}1}$ is utilized instead of *q*_2_. Here *L*_c_, is defined as $\sigma _{{{{\mathrm{c}}}}1}/\sigma _{{{{\mathrm{c}}}}2}$, with $\sigma _{{{{\mathrm{c}}}}1}\;(\sigma _{{{{\mathrm{c}}}}2})$ being the standard deviation of $q_{{{{\mathrm{c}}}}1}\;(q_{{{{\mathrm{c}}}}2})$, respectively. Figure 3b (left axis) demonstrates that this differential compensation cancels the channel offsets (details to the data analysis can be found in Section 10 in the SI). Moreover though, the benefit of our method is better revealed when comparing the standard deviation of the simple difference signal and that of our method, with $\sigma _{{{{\mathrm{q}}}}_1 - {{{\mathrm{q}}}}_2 - {{{\mathrm{q}}}}_{{{{\mathrm{avg}}}}}} = 33.3\;{{{\mathrm{mW}}/{\mathrm{m}}^2}}$ (yellow) and $\sigma _{{{\mathrm{q}}}} = 15.2\;{{{\mathrm{mW}}/{\mathrm{m}}^2}}$ (cyan) respectively. Such improvement in the fluctuation amplitude can be attributed to the reduction in the common-mode variation of the two channels (temperature or flow rate variations), as can be seen in Fig. 3b (left axis).

Subsequently, the same differential compensation based on $L_{{{\mathrm{c}}}},\;\bar q_{{{{\mathrm{c}}}}1},\;\bar q_{{{{\mathrm{c}}}}2}$ as determined in the calibration phase was used throughout for the correction of the raw heat flux data, e.g., upon the addition of bacteria as shown in Fig. 3c, d. Although, very hard to distinguish from the raw heat flux sensor output *q*_b1_ shown in Fig. 3c, after differential compensation, a clear exponential increase of the differential heat flux $q_{{{\mathrm{b}}}} = q_{{{{\mathrm{b}}}}1} - q_{{{{\mathrm{b}}}}2}^ \ast$ can be seen in Fig. 3d. This illustrates the merits of the differential compensation method and substantiates the use of two heat flux sensors.

After a sterilization step, the experiment was repeated, as shown in the Fig. S13 in Section 11 in the SI, this time reversing the function of the sensing and control sensor. There we observed a similar increase in heat flux upon addition of bacteria. At the beginning of the second experiment, the two heat flux sensors responded differently to the thermal stabilization step affecting the efficiency of the correction function applied to the raw data, as shown in detail in Fig. S13 in the SI.

### Determination of the single bacterium thermal power in the exponential phase

**Fig. 4:**
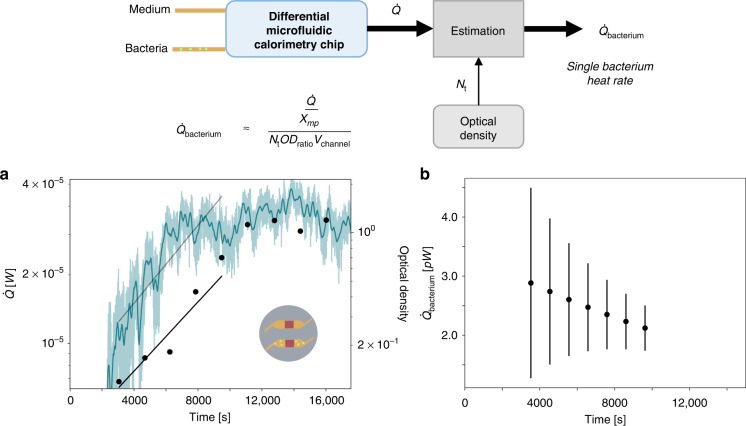
Estimation of the average thermal power produced by single E. coli bacterium. **a** $\dot Q = q_{{{\mathrm{b}}}}A_{{{{\mathrm{sensor}}}}}$ is the difference in thermal power measured in the differentially compensated microfluidic calorimetry chip, where *q*_b_ is the data from Fig. 3d (left axis). The raw data are shown in the lighter color and a 200-point moving average with a darker color line, and the fit is shown in dark gray. (right axis) The OD measurement is shown black with the exponential fit in a black line. **b** Estimated thermal power of a single bacterium from $\dot Q$ and optical density *N*_t_, where the heat transfer fraction *χ*_mp_ is determined via microcalorimetry (see Fig. 2). The extraction of OD_ratio_, as shown in Fig. S14 in Section 12 of the SI, and the determination of the error bars shown in (**b**), as shown in Section 6 in the SI

Figure 4b shows average thermal power produced by single bacterium calculated via:

3 $$\dot Q_{{{{\mathrm{bacterium}}}}} = \frac{{\dot Q/\chi _{{{{\mathrm{mp}}}}}}}{{N_{{{\mathrm{t}}}}{\rm{OD}}_{{{{\mathrm{ratio}}}}}V_{{{{\mathrm{channel}}}}}}}$$

where $\dot Q = q_{{{\mathrm{b}}}}A_{{{{\mathrm{sensor}}}}}$ is the compensated thermal power difference during the exponential growth phase of the bacteria (with *A*_sensor_ as the overlap surface area of the heat flux sensor and the microfluidic channel below it), *χ*_mp_ is the heat transfer fraction determined during a methyl paraben calibration experiment, *N*_t_ is the optical density measured during the exponential growth phase, OD_ratio_ is the conversion from optical density to bacteria/ml as described in Section 12 in the SI, and *V*_channel_ is the volume of the microfluidic channel. We determined the period of the exponential growth phase, $N_{{{\mathrm{t}}}} = N_0e^{{{\mu }}_{{{{\mathrm{OD}}}}}{{{\mathrm{t}}}}}$ by the OD measurement and extracted the corresponding exponential heat increase in the same time period, $\dot Q = \dot Q_0e^{{{\mu }}_{{{{\dot{\mathrm Q}}}}}{{{\mathrm{t}}}}}$. Comparing the rate of increase between the OD, *μ*_OD_, and the heat, *μ*_Q_, we find that their ratio, *μ*_Q_/*μ*_OD_, is 1.4 in the experiment shown (Table 1).

**Table 1:** Summary of extracted values

System	*μ*_OD_ [s^−1^]	*μ*_Q_ [s^−1^]	*χ*_mp_ [%]	*Q*_bacterium_ [pW]
Sensor 1: Sensing	2.81 ∙ 10^−4^	1.97 ∙ 10^−4^	77 ± 8	1.3–4.5

Exponential bacterial growth rate from the optical density *μ*_OD_ [s^−1^], rate of heat increase $\mu _{{{{\dot{\mathrm Q}}}}}\left[ {{{{\mathrm{s}}}}^{ - 1}} \right]$, the heat transfer fraction of the microfluidic chip *χ*_mp_, and the single bacterium thermal power $\dot Q_{{{{\mathrm{bacterium}}}}}$. Further discussion of the data to be found in Section 11 of the SI

The thermal power of a single bacterium is expected to stay constant in the exponential growth phase, however, Fig. 4b shows a decreasing trend in the single bacterium thermal power with increasing time. This could be due to a change from real exponential growth to a transition to the stationary phase or the switch between nutrient sources which is known to occur in LB media^39^. On the other hand, Alklint et al.^17^ showed a concave curve for thermal power over time for the bacterial growth, which also indicates a non-constant single bacterium thermal power over time. The doubling time of the *E. coli* for the experiment is determined as 42 min (see Section 3 of the SI for detailed doubling time determination). The range of single bacterium thermal power determined with our microfluidic chip of 1.3–4.5 pW (Fig. 4b) is in the range of values previously found in literature for *E. coli* in LB media of 3.5 pW^40^.

### Determination of the limit of detection of the system

As shown in Fig. 3b, the noise in the differential heat flux data is significantly reduced from $\sigma _{{{\mathrm{q}}}} = 34\;{{{\mathrm{mW}}/{\mathrm{m}}^2}}$ to $\sigma _{{{\mathrm{q}}}} = 15\;{{{\mathrm{mW}}/{\mathrm{m}}^2}}$ upon averaging over 200 data points (sampling frequency ≈ 0.7 Hz). The time interval of averaging was chosen to be significantly smaller than the expected doubling time of bacteria $\tau _{{{{\mathrm{bacteria}}}}} \gg {\Delta}t_{{{{\mathrm{averaging}}}}}$. We determined a sensitivity in units of thermal power per optical density of $6.8 \times 10^{ - 14}{{{\mathrm{W}}}}/{{{\mathrm{bacteria}}}}$ $\left( {0.73\;\frac{{{{\mathrm{W}}}}}{{{{{\mathrm{m}}}}^2}}/{{{\mathrm{OD}}}}} \right)$, and a limit of detection of 2 × 10^7^ bacteria (0.021 OD) in our system, as described in detail in Section 13 of the SI. In comparison, commercial microcalorimeters, achieve a limit of detection corresponding to 10^4^ active microbial cells^41^. In units of thermal power density, the limit of detection was determined to be 1707 W/m^3^, assuming a thermal power of a single bacterium of 3.5 pW^40^ with the considered channel volume of 46 μl. As a comparison, a biofilm contains ~15% active bacteria^42^, and assuming a bacterial thermal power of 3.5 pW and bacterial volume of 1.49 μm^3 ^^43^, the thermal power density of a bacterial biofilm can be estimated as 350,000 W/m^3^ (see Section 14 in the SI for the determination of thermal power density). As this is three orders of magnitude higher than the limit of detection of our microfluidic chip, this demonstrates the potential of our microfluidic chip for the early detection of the onset of biofilm growth.

## Conclusions

This work presents a first demonstration of the integration of a differential heat flux sensing system onto a microfluidic chip. This new approach enables access to calorimetric information in microfluidic channels for investigating micro scale metabolic changes in cells. Previously, the design of microcalorimetric chips has focused on the thermal optimization of the system rather than microfluidic aspects. This may explain why most chip calorimeters have been employed in monitoring chemical or biochemical reactions—for example, hydration of ethanol or propanol^44^, glucose oxidation by glucose oxidase^34^, or denaturation of protein^33^—and less on cell metabolism.

We show the measurement of thermal activity of *E. coli* bacteria from the lag phase throughout the exponential growth phase to the stagnation of growth. We calibrate the heat transfer fraction using an exothermic reaction with methyl paraben. The experimentally determined heat transfer fraction *χ*_mp_ of 77 ± 8% is comparable to the simple lumped element model we propose, with a heat transfer fraction of 73%. We apply a differential compensation data analysis method to extract the thermal power increase during the growth of bacteria. The amount of heat produced by such communities is a direct proxy for their metabolic efficiency, and this measurement can thus be used to investigate questions concerned with ecology and evolution. We are able to extract the thermal power of a single bacterium between 1.3 and 4.5 pW, which is comparable to the single *E. coli* thermal power reported in literature in LB media of 3.5 pW^41^. A limit of detection of 0.21 OD (2 × 10^7^ bacteria), corresponding to 1707 W/m^3^ was determined. To set things in perspective with a simple example, the determined LOD is orders of magnitudes below the power density of a biofilm with 15% active *E. coli* bacteria. Thus, our differential calorimetric chip may find use in the detection of biofilm formation, which is useful for the early detection and treatment of bacterial infections on implant surfaces.

Our approach prioritizes integration flexibility as opposed to the thermal stability focus of microcalorimetric chip systems found in literature. In order to achieve this flexibility, we eliminated the often employed PID temperature controllers, microheaters, and thermally stable vacuum/air insulation layers. As such, a tradeoff between the accuracy and the system integration flexibility was made. Nonetheless, we were able to determine the thermal power of an *E. coli* bacteria in good agreement with previous literature values. The key to achieving a useful accuracy was adopting a differentially compensated heat flux measurement. As opposed to using several temperature sensors, we opted for heat flux sensors, allowing for the direct measurement of the thermal power produced in the microfluidic channel, while also reducing the amount of sensors needed. The differential measurement using two heat flux sensors enables the common mode rejection of the background thermal fluctuations. Further work should explore if this common mode rejection is enough to allow for the system to measure in laboratory ambient conditions, as opposed to thermally controlled chambers.

The core of the setup is a simple, easy to modify, and fabricate PDMS microfluidic chip with integrated commercial heat flux sensors. The ease of production of the presented system and its adaptability could benefit researchers as the microfluidics can be rapidly modified to fit a specific experiment while the sensor part will remain the same and just needs to be mounted, allowing for a disposable microfluidics system for the use in diagnostics. In addition, our design allows for the heat flux sensors used to be retrieved from above the microfluidic structure allowing for reuse, keeping each microcalorimetric system variation cost to a minimum. With minimal adjustments, our PDMS-based microcalorimetric design could be utilized in the future to extend existing lab-on-chip systems^7,45–47^, or investigations of drug delivery efficiency^48,49^, research in microbiology on the influence of effects of environmental switches, metabolic changes, cell growth, gene expression and ageing of cells, and exothermic chemical reactions^50,51^.

## Materials and methods

### Preparation and handling of E. coli MG1655

Prior to each bacterial experiment, all the equipment was thermally equilibrated overnight within the incubator at 37 °C. The bacterial serum was parallelly prepared and grown in LB media at 37 °C overnight from a single colony on a petri dish with LB agar. The bacteria were continuously quantified using an optical density meter (OD meter) periodically by collecting a liquid sample at the outlet of the microfluidic chip every 25 min set to a wavelength of 600 nm. The *MG1655 E. coli* doubling time was determined to be 42 min (about double the time of the OD sampling), as described in detail in Section 3 in the SI.

Measurements were performed in a 37 °C environment of a thermal incubator for optimal bacterial growth, and the container with the bacteria was aerated using a magnetic stirrer at 200 rpm. For minimal bacterial adhesion in the tubing, 0.5 ml of 1:100 tween20 was added to 50 ml LB media. In total, 1 ml of *MG1655 E. coli* serum was then added to the prepared LB media and tween20 mixture. Prior to being measured by the heat flux sensors in the microfluidic chip, the bacteria traveled for 5–10 min in unaerated tubing. The time difference between the OD measurement and the heat measurement was calculated using flow rate, tubing cross section and the travel distance in the Polytetrafluoroethylene (PTFE) tubing. We showed a minimal difference in the exponential growth phase between the aerated container in which bacteria is growing and the outlet after anaerobic growth, as also shown experimentally in Section 15 in the SI. This eliminates a source of systematic error in bacterial population and heat measurement^52^. Following the bacterial experiment, the microfluidic chip was sterilized with 70% ethanol and flushed with sterile deionized water for multiple hours in preparation for the subsequent calibration experiment with methyl paraben.

### Characterization of temperature and heat flux sensors

Prior to each experiment at 37 °C, the equipment was thermally equilibrated for overnight within the incubator or oven at 37 °C. Measurements were performed in a 37 °C environment of a thermal incubator for optimal bacterial growth, however without stirring. In total, 0.5 ml of 1:100 tween20 was added to 50 ml LB media. In total, 1 ml of bacteria was then added to the LB and tween20. A microfluidic chip with two channels 320 µm height with two embedded heat flux sensors was used. PTFE tubing with ID of 0.012 in cut to a length of 0.3 m from Cole Palmer (06417-11) was used to transport the media without bacteria and the media with *MG1655* to the microfluidic channel. A peristaltic pump (Instech P720) was used to transport the fluids from the reservoir using peristaltic tubing with an ID of 0.02 (P720/TS-020S Instech Laboratories) and length of 0.3 m. 21G Sterican needle of 0.12 m length was used to draw the liquids out of the containers and connect to the tubing. To improve thermal stability, the microfluidic chip was placed in a PMMA box with two 4 mm PMMA layers separated by a 12 mm layer of air between the walls. Following the bacterial experiment, the microfluidic chip was sterilized with 70% ethanol and flushed with sterile deionized water for multiple hours in preparation for the subsequent calibration experiment with methyl paraben.

### Methyl paraben calibration experiments

#### Methyl paraben microfluidic calorimetric chip experiment

A similar setup to Fig. 1a was used for the methyl paraben microfluidic calorimetric chip experiment, as illustrated in Fig. S1 in Section 2 of the SI. The calibration experiment was performed according to previous investigations for flow-through microcalorimeters by O’Neill et al.^36^ with modifications. In total, 20 ml of 0.5 M NaOH, Sigma-Aldrich, was mixed with 152.32 mg methyl-4-hydroxy-benzoat (methyl paraben), Sigma-Aldrich ≥ 99.0% ReagentPlus®. The time at which the methyl paraben and NaOH were mixed was tracked, as that is the time point to which the experiments are compared to each other with. We performed the experiment at 37 °C in an oven (Vötsch 4006) and waited 5–10 min for the thermal stabilization after the mixing of the components. We used DI water in our reference channel to measure the background noise from the oven and the noise of the flow from the peristaltic pump. As the thermally stable state, we used the end of the dataset to determine the reference 0 of the system, as is common for microcalorimetry experiments.

#### Methyl paraben microcalorimetric experiment

In total, 10 ml of 0.5 M NaOH, Sigma-Aldrich, was mixed with 76.66 mg methyl-4-hydroxy-benzoat (methyl paraben), Sigma-Aldrich ≥ 99.0% ReagentPlus®^36^. The time at which methyl paraben and NaOH were mixed was tracked and used for the comparison to the methyl paraben calibration experiment of the microfluidic calorimetric chip. The experimental setup was performed with a TAMII nano (Waters/TA, Delaware, USA). DI water was used as the reference, similarly to the methyl paraben calibration experiment in the microfluidic chip.

### Microfluidic chip fabrication

The structures were drawn using KLayout software and transferred on a 5 inch soda lime mask using a DWL2000 (Direct Write Laser Heidelberg Instruments) with laser wavelength of 413 mm. The Cr on the mask was then etched for 40–50 s in Chrome ETCH N1, rinsed and then placed in Technistrip P1316 for 10 min. SU-8 2150 was patterned on a 4 in silicon wafer using a Karl Suss MA6 Mask Aligner. SU-8 2150 was applied in excess to a silicon wafer and then spin coated for 30 s at 500 rpm with 200 rpm/s to spread the photoresist and then 30 s at 1000 rpm with 300 rpm/s for the final thickness. The wafer was soft baked for 9 min at 70 °C, then ramped to 100 °C and baked for 98 min. Followed by an exposure step of 400 mJ/cm^2^ corresponding to 55.6 s exposure. The wafer was then bake for 5 min at 65 °C and then ramped to 95 °C and baked for 25 min. Then, the wafer was developed for 25 min in Mr Dev 600 (micro resist technology GmbH). Following the development, the wafer was rinsed in isopropanol and subsequently in deionized water. Lastly, the wafer was hard baked at 150 °C for 5 min. The structures were diced in appropriate pieces which were then used for PDMS casting.

PDMS (Sylgard 184) was mixed vigorously for 4 min in a 1:10 ratio between elastomer and crosslinker. After 40 min vacuum desiccation or until no visible bubbles. Using metal spacers, the thickness of the PDMS layer between the sensor and the bacterial channel was defined as 0.15 mm and cured at 80 °C for 3 h. The heat flux sensors were then secured on top of the cured PDMS layer and embedded by pouring another layer of PDMS with a defined thickness and cured again at for 80 °C for 3 h. After curing, the PDMS structure was cut out using a scalpel and fused with a glass slide using plasma (Diener Oxygen Plasma Asher). A 20G needle was used to punch out the inlet and outlets of the microfluidic chip. The plasma was applied on both the glass slide and the PDMS at 0.8 mbar with 100 W in a O_2_ environment for 90 s. The pieces were then directly fused together within 60 s of the plasma treatment and placed at 80 °C for 15 min.

### Data analysis

The two GreenTeg gSKIN XP 26 9C heat flux sensors were characterized using two Keithley 2000 multimeters. The temperature sensor PT1000 was measured using a Keithley 2400 sourcemeter, and the temperature value was directly applied to convert the voltage to heat flux. The python package “visa” from “pyvisa” was used for the control (read, write, query) and real time readout of the sensors. The voltage output of the heat flux sensors was tracked and the resistance of the temperature sensor. The heat flux sensors were embedded in PDMS of a microfluidic chip.

## Supplementary information

Supplementary Information

## Data Availability

The data are available upon request from the author.
