# Correction: Enabling direct microcalorimetric measurement of metabolic activity and exothermic reactions onto microfluidic platforms via heat flux sensor integration

**DOI:** 10.1038/s41378-024-00684-7

**Published:** 2024-05-27

**Authors:** Signe L. K. Vehusheia, Cosmin Roman, Olivier Braissant, Markus Arnoldini, Christofer Hierold

**Affiliations:** 1 Micro and Nanosystems, Department of Mechanical and Process Engineering, ETH Zurich, Tannenstrasse 3, 8092 Zurich, Switzerland; 2 Center of Biomechanics and Biocalorimetry, Department of Biomedical Engineering, University of Basel, Hegenheimermattweg 167C, 4123 Allschwil, Switzerland; 3 Laboratory for Food Immunology, Department of Health Sciences and Technology, ETH Zurich, Otto-Stern-Weg 3, 8093 Zurich, Switzerland

**Keywords:** Microfluidics, Sensors, Biosensors

Correction to: *Microsystems & Nanoengineering*

10.1038/s41378-023-00525-z published online 09 May 2023

After publication of this article [1], we have identified typographical errors in the published version that require correction, the necessary changes are as follows:

**Correction 1:**

In the abstract and page 8 of the manuscript 2 × 10^7^ bacteria should be 2 × 10^7^ bacteria/mL. Similarly in table S11 in the Supplementary Information #bacteria should #bacteria/mL.

**Correction 2:**

In the Supplementary Information in line 188 ${\dot{Q}}_{{down}}$ should be $\,{\dot{Q}}_{{up}}$ in the equation, and in line 189 ${\dot{Q}}_{{down}}$ should be $\,{\dot{Q}}_{{up}}$.
