# Supplementary material for: Enabling direct microcalorimetric measurement of metabolic activity and exothermic reactions onto microfluidic platforms via heat flux sensor integration
Source: Microsyst Nanoeng. 2023 May 9;9:56. doi: 10.1038/s41378-023-00525-z (PMC10169645; doi:10.1038/s41378-023-00525-z)
Supplement: Supplementary file 1 [file 41378_2023_525_MOESM1_ESM.docx]

Supplementary Information

1. Key Performance Metrics

Table S1 is an overview over the main key performance metrics of the differential microfluidic calorimetric chip. Some of the values are derived from the system data analysis and some are from the lumped element model analysis.

Table S1 An overview over the key parameters of the differential microfluidic calorimetric chip: **Name**

Name	Value	Description
Limit of detection	$1707 \frac{W}{m^3}$	System data analysis elaborated on in section 13
Standard deviation of noise	$0.015 \frac{W}{m^2}$, $15\,nW$	System data analysis elaborated on in section 13
Sensitivity	$0.73 \frac{W}{m^2}/OD$	System data analysis elaborated on in section 13
Time constant τ	$9\,s$	Lumped element model analysis described in section 7: the largest time constant of all the materials
Most important thermal conductivity k of $PDMS$	$0.16 \frac{W}{mK}$	Lumped element model analysis described in section 7: the material with lowest thermal conductivity in the system is PDMS
Total thermal conductivity k	$0.17 \frac{W}{mK}$	Lumped element model analysis described in section 7: total calculated thermal conductivity of the microfluidic system
Thermal capacitance	$0.25 \frac{J}{K}$	Lumped element model analysis described in section 7: the material with the largest thermal capacitance affecting the measurement is PMMA
Heat transfer fraction χ_{mp}	Modelled: 73%<br>Experimental: $77\% \pm 8\%$	System data analysis elaborated on in section in Fig. 2 a)

2. Methyl Paraben Experimental Setup

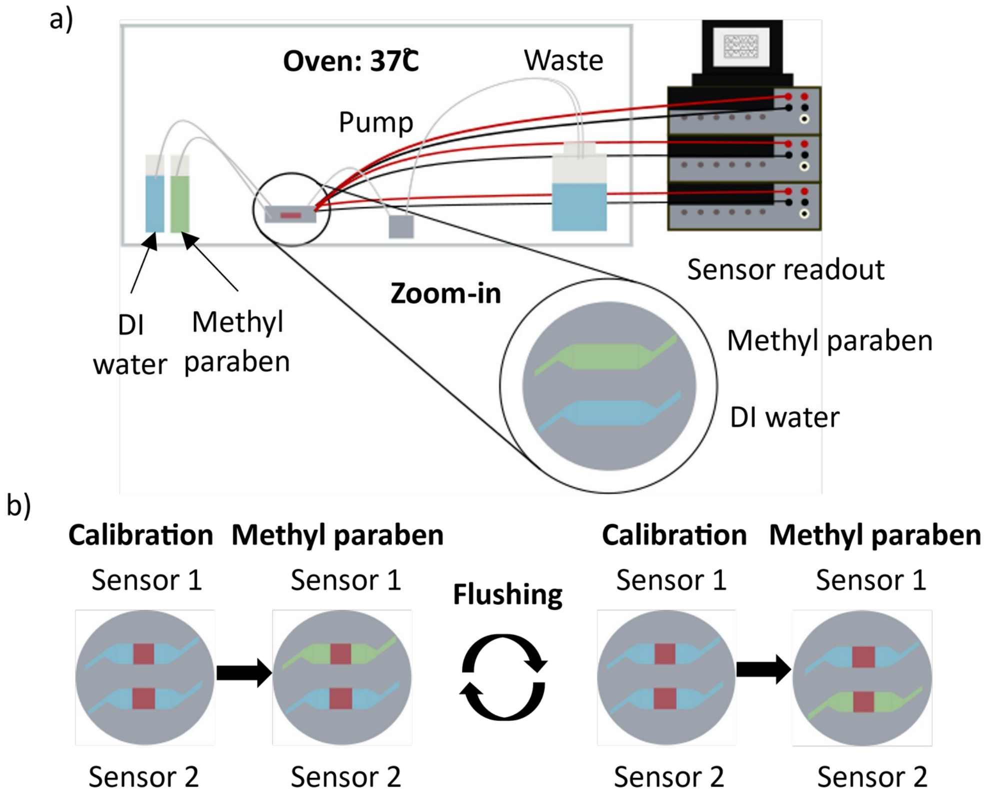

Figure S1 Experimental setup for methyl paraben experiment with microfluidic calorimetric chip: *Methyl paraben experimental setup within a* $37^{o}C$ *oven. Note that the environment is not the same as during the bacterial experiment as we used an oven instead of an incubator.*

The experimental setup was in an oven at $37^{o}C$ (Vötsch 4006) and a peristaltic pump (Instech P720) was used to control the flow rate of the liquid through the channels at $98.8\frac{\mu L}{min}$ (for the experimental results shown in Fig. S10) and $67.5\frac{\mu L}{min}$ (for the experimental results shown in Fig. S11). In this experimental setup the PMMA box was not used for thermal stabilization. For the control channel we used DI water with the same flow rate as the other channel with methyl paraben. The experiment was repeated with opposite sides after a DI water flushing step of 1-2 hours, similarly to the sterilization step in the bacterial experiment.

3. Determination of the Doubling Time of *E. coli*

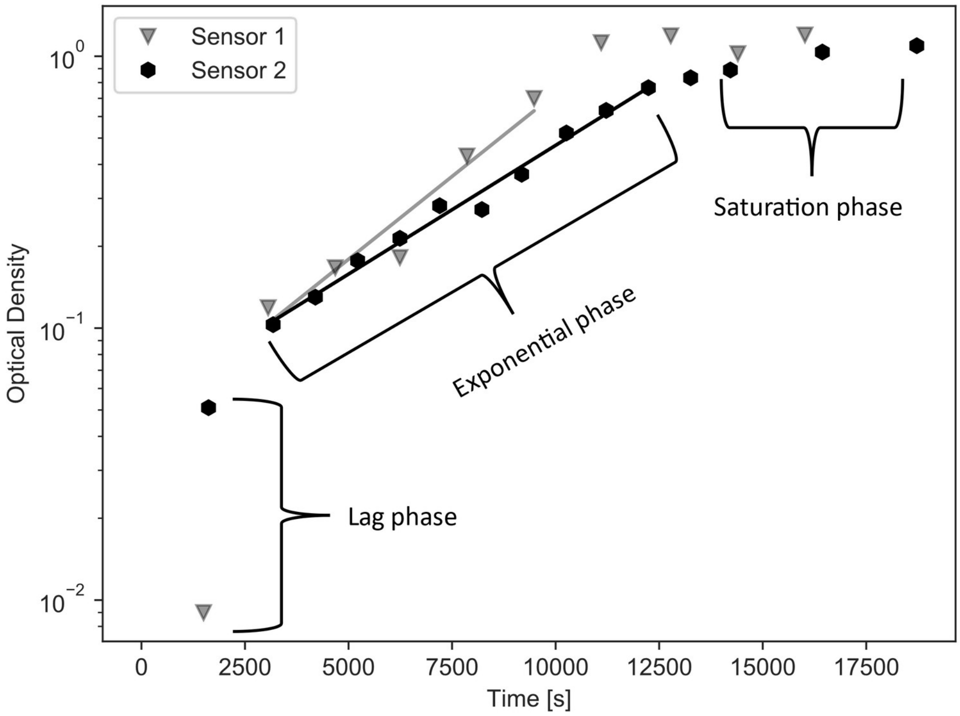

Figure S2 Determination of E. coli doubling time: *The bacterial growth curves for both the experiments as also plotted in Fig. S13 a, b). Experiment named Sensor 1 corresponds to the optical density data shown in Fig. 4 a) in the main text, where the bacterial growth was within the channel of Sensor 1. The data names Sensor 2 shows the bacterial growth upon sterilization and bacterial growth within the channel of Sensor 2. Three phases can be identified: lag phases, exponential phase, and the saturation phase.*

First, the data was fit to a linear function in the semi-logarithmic (as shown above), and the slope was determined in the exponential growth phase. The doubling time G can be calculated as:

$$G=\frac{(t_1-t_0)}{generations}=\frac{(t_1-t_0)}{3.3\log\left(\frac{n_1}{n_0}\right)}$$

The formula for the doubling time can be derived from:

$$n_1 = n_0 \times 2^{generations}$$

$$\log(n_1) = \log(n_0) + generations \log(2)$$

$$generations = ¿¿$$

$$generations = 3.3\, log\left(¿\frac{n_1}{n_0}\right)¿$$

Table S2 The calculated doubling time and the slope of the exponential growth

System	Doubling time G	Slope μ_{OD}
Sensor 1	41.65 min	$2.81 \cdot 10^{-4}\ \frac{1}{s}$
Sensor 2	56.08 min	$2.18 \cdot 10^{-4}\ \frac{1}{s}$

The variation could be due to the anaerobic to aerobic changes and the different flow rates of the system leading to different resident times (10-25 min) within the anaerobic conditions of the tubing in the experiment.

4. Microcalorimetric Experiment

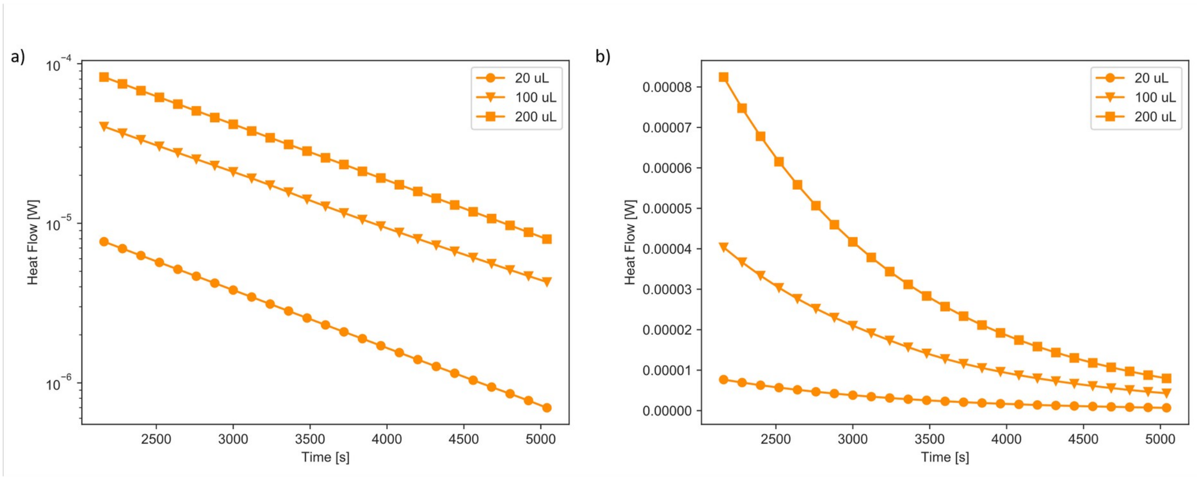

Figure S3 Microcalorimetric experiments: *a) Microcalorimetric data for three different volumes: $20\,\mu L$, $100\,\mu L$, and $200\,\mu L$ at $37^{o}C$. b) Same data in semi-log plot.*

The raw data was fit to a linearized function:

$$x = \ln(a) - bt$$

Table S3 Extracted values from the fit with the respective covariance: *Extracted slope from literature by O'Neill[5] in comparison.*

	**a**	**b**
	Intercept [W]	Slope [$\frac{1}{s}$¿
$20\,\mu L$	$4.63 \cdot 10^{-5}$	$-8.32 \cdot 10^{-4}$
Error (covariance)	$9.36 \cdot 10^{-8}$	$2.089 \cdot 10^{-13}$
$100\,\mu L$	$2.22 \cdot 10^{-4}$	$-7.859 \cdot 10^{-4}$
Error (covariance)	$2.82 \cdot 10^{-6}$	$7.159 \cdot 10^{-12}$
$200\,\mu L$	$4.73 \cdot 10^{-4}$	$-8.101 \cdot 10^{-4}$
Error (covariance)	$7.99 \cdot 10^{-7}$	$1.687 \cdot 10^{-13}$
Literature[5]	---	$-7.75 \cdot 10^{-4}$

The slope observed in literature corresponds to the slopes measured using the microcalorimetric system. The different y-axis intercepts of the microcalorimetric experiment scale with the initial volumes of the samples (1, 4.8, and 10.2), as it indicates the heat of the system.

5. Data Analysis of Raw Methyl Paraben Data

Fig. S4 and S5 show, similar to the *E. coli* experiment, that the data from the methyl paraben experiments underwent the same differentially compensated data analysis method. The determined differential heat was subsequently used in comparison to the microcalorimetric experiments with methyl paraben. We extracted the y-axis intercept according to the fit shown in Fig. S4 e) and S5 e). Fig. S13 show the differentially compensated measured thermal power measured upon addition of methyl paraben (as shown in Fig. S4 e) and S5 e)) in comparison to the microcalorimetric measurements. The heat transfer fraction χ_{mp} was determined as described in the main text as $77\%\pm8\%$ for Sensor 1 and $52\%\pm9\%$for Sensor 2.

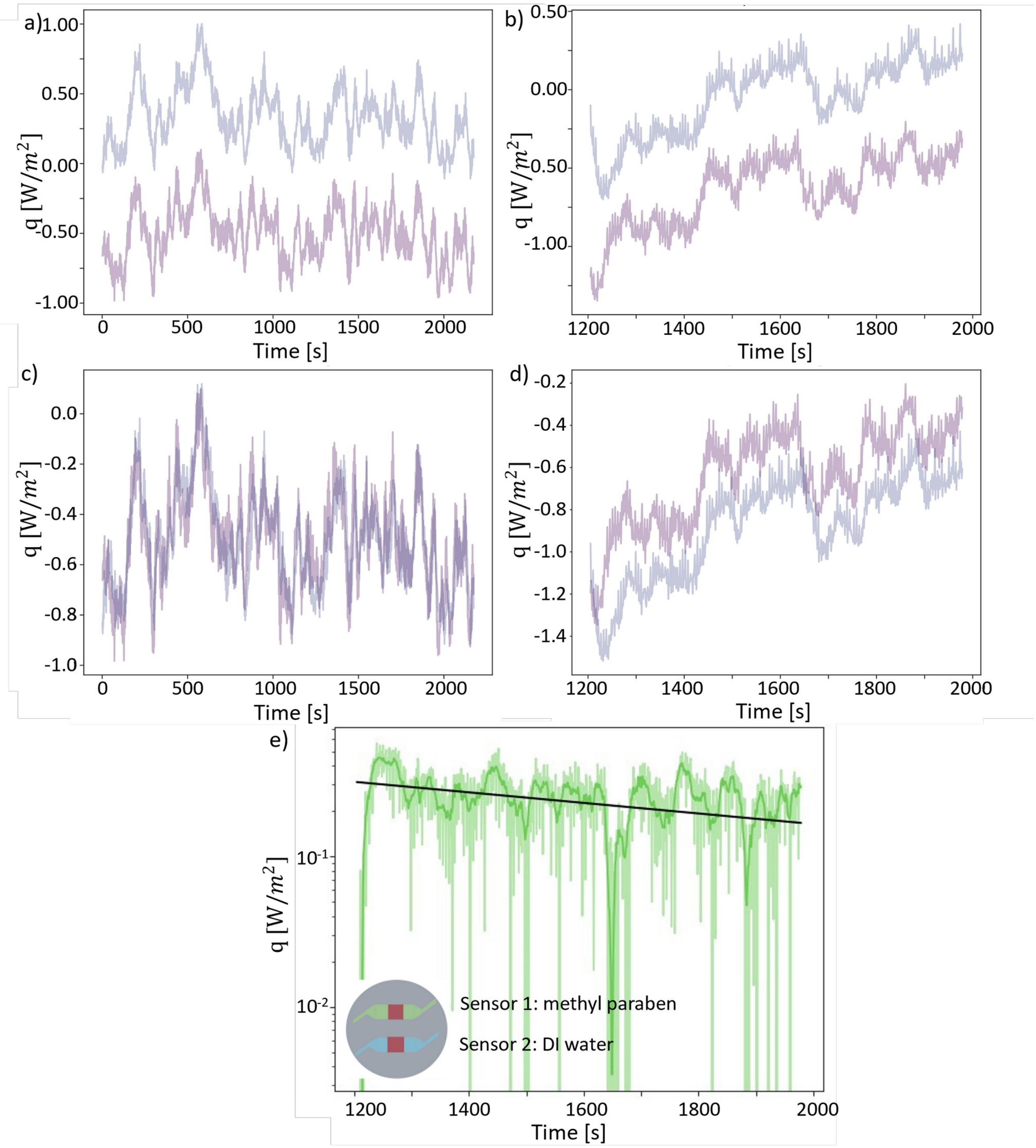

Figure S4 Methyl paraben experiment with microfluidic calorimetric chip: a, c) *Calibration phase where both sensors contain DI water only, and* ***c), d)*** *Methyl paraben experiment where the sensing channel contains methyl paraben mixed with NaOH. In* ***c, d)*** *the correction of* ***a, b)*** *is shown, with* ***c)*** *specifically showing the overlap between the two sensors during the calibration phase. In* ***a-d)*** *the raw data is shown with purple being consistently the methyl paraben sensing sensor (Sensor 1) and blue being consistently the DI water compensation sensor (Sensor 2).* ***e)***

The differentially compensated heat flux upon addition of methyl paraben mixed with NaOH (as described for the E. coli experiment) of the raw data shown in the lighter color, and for a 10 point moving-average with a darker color line. The black line indicates the exponential fit.

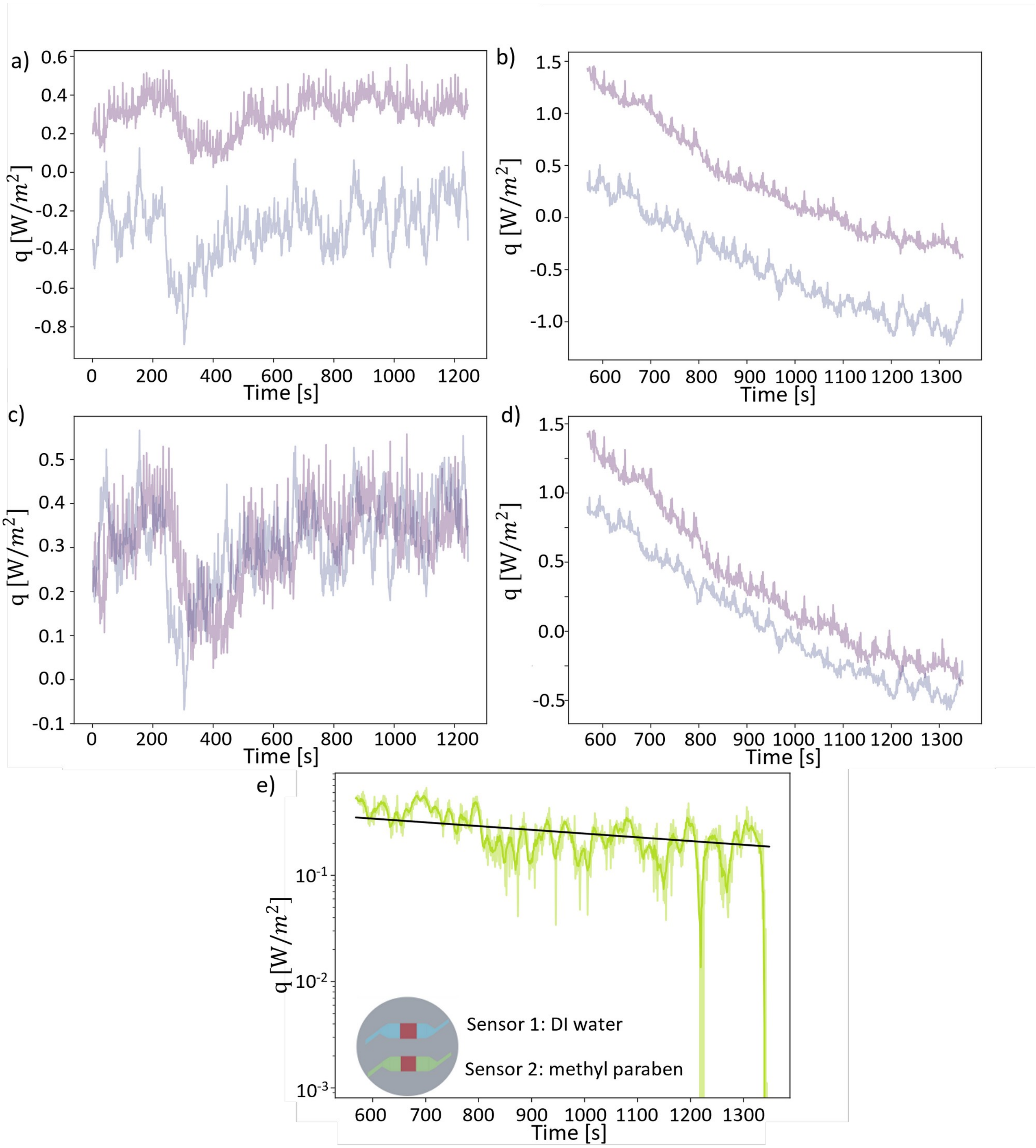

Figure S5 Methyl paraben experiment with microfluidic calorimetric chip: a), c) *Calibration phase where both sensors contain DI water only, and* ***c), d)*** *Methyl paraben experiment where the sensing channel contains methyl paraben mixed with NaOH. In* ***c, d)*** *the correction of* ***a, b)*** *is*

*shown, with **c)** specifically showing the overlap between the two sensors during the calibration phase. In **a-d)** the raw data is shown with purple being consistently the methyl paraben sensing sensor (Sensor 2) and blue being consistently the DI water compensation sensor (Sensor 1). **e)** The differentially compensated heat flux (as described for the E. coli experiment) of the raw data shown in the lighter color, and for a 10 point moving-average with a darker color line. The black line indicates the exponential fit.*

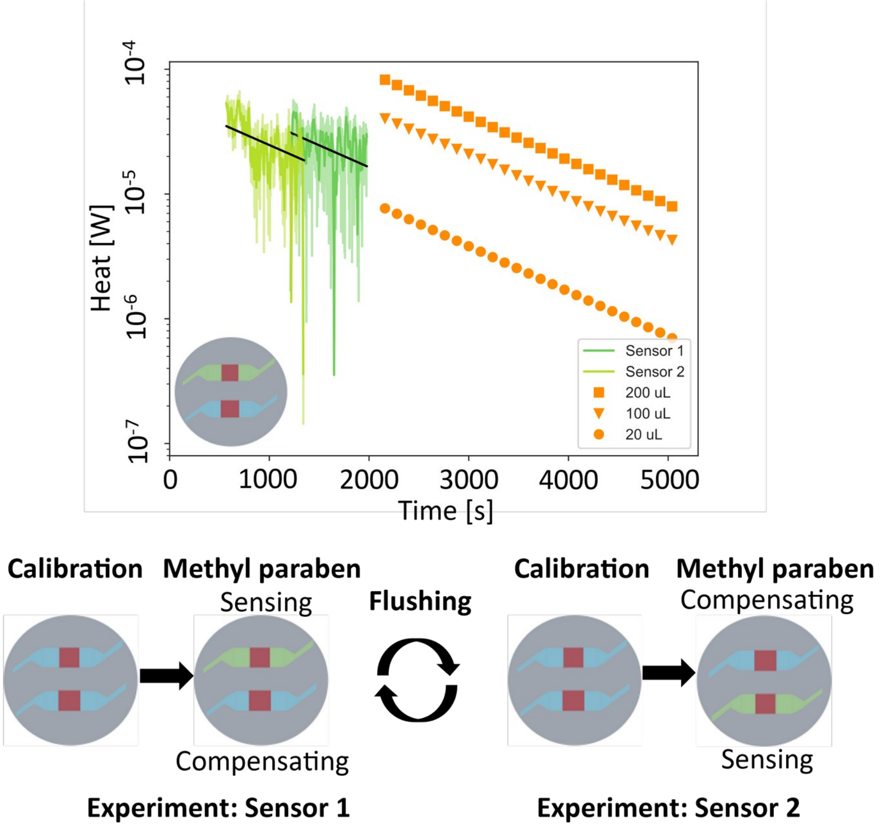

Figure S6 Comparison between microcalorimetric and microfluidic calorimetric experiments: *Determination of the heat transfer fraction χ_{mp} in both experimental configurations. Similar to Fig. 2 a) in the main text with the additional experiment for the second experimental configuration where the compensating and sensing sensor were switched. The data labelled Sensor 1 had methyl paraben in Sensor 1, and the data labelled Sensor 2 had methyl paraben in Sensor 2.*

6. Error Propagation for the Single Bacterium Thermal Power

To determine the error propagation of the acquired data we determine the errors of the exponential fits. We exponentially fit the data at 4 different experimental values: the microcalorimetric experiment of methyl paraben, the methyl paraben calibration of the microfluidic chip, the difference of the heat produced by the bacterial growth in the exponential growth phase (determined by the optical density), and the optical density measurement in the exponential growth phase, as illustrated below.

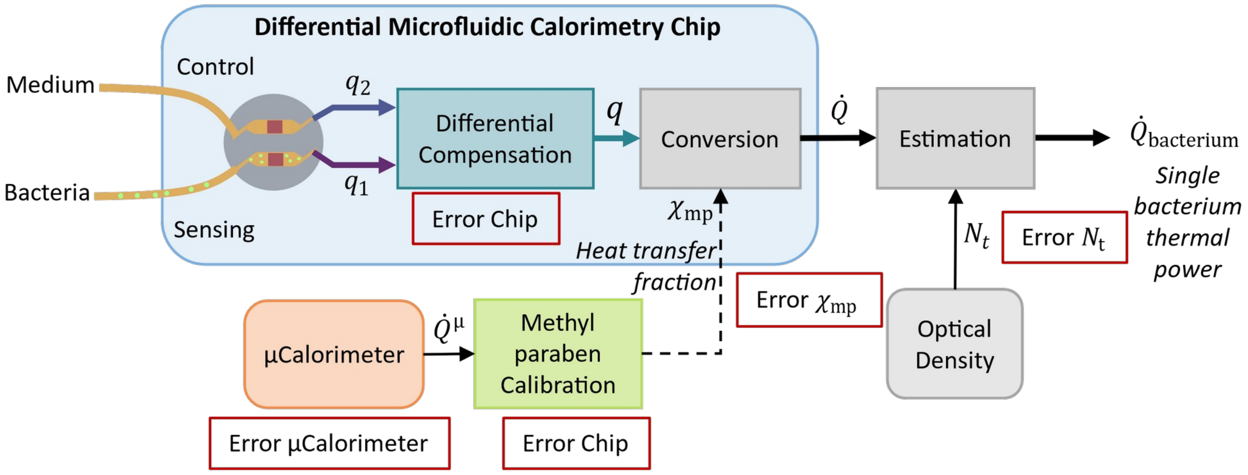

Figure S7 Schematic of error propagation: *Modified overview over the experiments and the source of errors for the final error of the heat of a single bacterium.*

The respective errors in the exponential fits of the methyl paraben experiments with the microcalorimeter using the Python curve_fit function. The value *a* shown in Table S4 is the intercept with the y axis at time = 0 s. We determined the standard deviation of the residual between the fit and the heat data (averaged over 10 data points) in the microfluidic chip and used this as the error.

Table S4 Overview over values and error used during the methyl paraben experiments:

Errors of the methyl paraben experiment $\delta\chi_{mp}$highlighted in bold.

**Value**	**Intercept with y axis (x=0)**	**Description**
Microcalorimetry 20 µL $\dot{Q}_0^{\mu/20\mu L}$	a = 4.6· 10^{-5} ± 9.4 · 10^{-8} W	For 20 µL
Microcalorimetry 20 µL scaled $\dot{Q}_0^{\mu}$	1.1· 10^{-4} ± 2.2· 10^{-7} W	Scaled to 46 µL
Sensor 1 methyl paraben $\dot{Q}_{m0}$	8.3 · 10^{-5} ± 8.8· 10^{-6} W	For 46 µL
Sensor 2 methyl paraben	5.5· 10^{-5} ± 9.2· 10^{-6} W	
**Value**	**Heat transfer fraction**	
Sensor 1 χ_{mp}	77 ± **8 %**	
Sensor 2 χ_{mp}	52 ± **9 %**	

Measurement by the microcalorimeter for 20 µL fit using an exponential (as shown in the previous section):

$$y=a\exp(¿-bx)-c¿$$

First, we scaled the system to 46 µL by multiplying by the ratio between 46 µL and 20 µL to the values of a. Then, we applied the error propagation formula while determining the heat transfer fraction of the system $\chi_{mp}=\dot{Q}_{m0}/\dot{Q}^{\mu}{}_0$.

$$\delta\chi_{mp}=\chi_{mp}\sqrt{¿¿}$$

The exponential growth phase of the bacteria was fit to an exponential function both the OD and the heat data:

$$\ln(¿y)=at+b¿$$

The respective values and errors were determined by investigating the exponential fit and using the standard deviation of the residual as the error as shown in Table S5. For the single bacteria thermal power of $\dot{Q}_{bacteria}=\frac{\dot{Q}}{\chi_{mp}}/N_t OD_{ratio}V_{ratio}$, the error is calculated as:

$$\delta\dot{Q}_{bacteria}=\dot{Q}_{bacteria}\sqrt{¿¿}$$

With considered errors in $\dot{Q}$, χ_{mp}, and N_t.

Table S5 Errors of the system during the bacterial experiment: *Errors shown for both experimental configurations of bacterial thermal power and OD as shown in Fig. S13.*

**Value**	**Error**	**Description**
OD 1 N_t	0.0464	Standard deviation during calibration
OD 2 N_t	0.0251	Standard deviation during calibration
Sensor 1 differential heat $\dot{Q}$	$5.45 \cdot 10^{-6}$	Error in the calibration phase (averaged 200 data points) W
Sensor 2 differential heat $\dot{Q}$	$2.26 \cdot 10^{-6}$	Error in the calibration phase (averaged 200 data points) W

Hereby, the error bars of both experiments as shown in Fig. S13 c) were determined.

7. Lumped Element Model

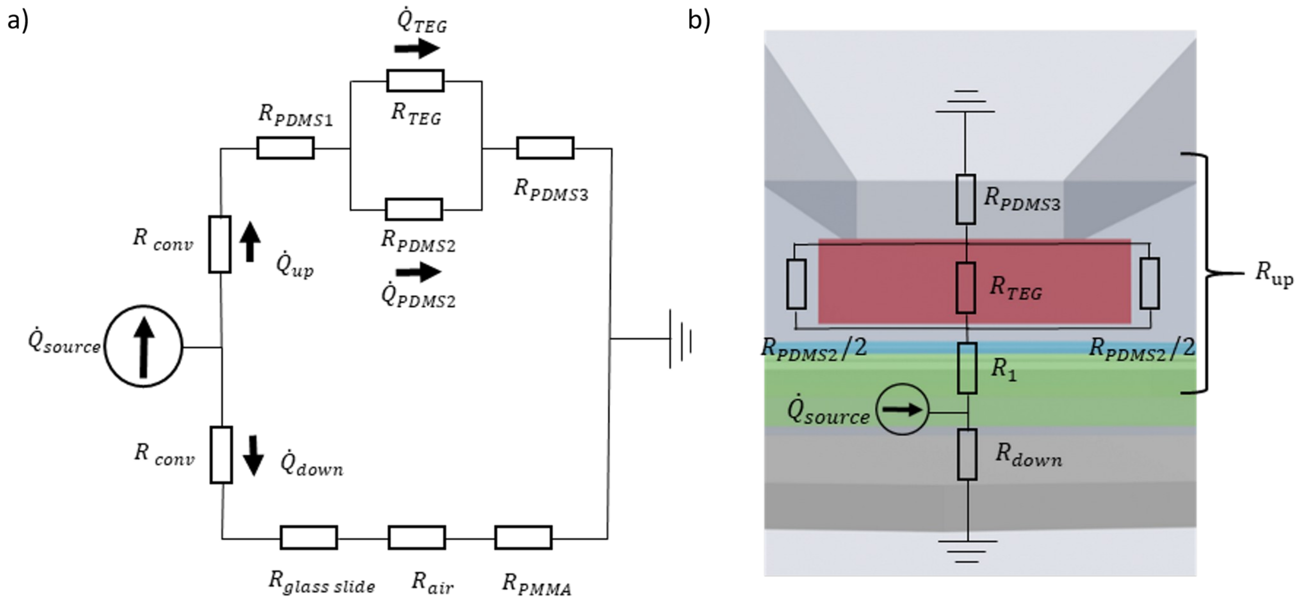

Figure S8 ***Schematic over the lumped element model:*** *a) Lumped element model of our system. b) Sketch showing the corresponding values of the lumped element model as also shown in Fig. 2 in the main text. With* $R_1 = R_{conv} + R_{PDMS1}$ *,* $R_{down} = R_{conv} + R_{glass} + R_{air} + R_{PMMA}$ *and* R_{conv} *being the convective heat transport.*

Using a lumped element model, we express the heat flux produced by the bacteria as the current source $\dot{Q}_{source}$. We assume that the heat flow has two pathways, either up $\dot{Q}_{up}$ towards the heat flux sensor or down $\dot{Q}_{down}$ away from the heat flux sensor. The pathways to the sides are neglected due to their smaller surface volume in comparison to the pathways upwards and downwards. The heat flowing upwards passes an initial PDMS layer separating the channel and the heat flux sensor. Furthermore, the surface region considered is larger than the surface of the heat flux sensor ($12\,mm \times 12\,mm$), which leaves a pathway of heat through PDMS in parallel to through the heat flux sensor R_{PDMS2}. Following the heat flux sensor, there is a layer of PDMS on top, R_{PDMS3}, underneath the copper block, which is not considered due to its low thermal resistance. The copper block is considered at a constant temperature, and thus considered to be on the pathway downwards $\dot{Q}_{down}$, we consider the glass slide $R_{glass\,slide}$, an air gap between the PMMA stand and the glass slide R_{air}, and the PMMA stand R_{PMMA}.

We apply a current divider twice to express the heat flow through the heat flux sensor $\dot{Q}_{TEG}$ over the total heat produced in the microfluidic channel $\dot{Q}_{source}$, which is the heat transfer fraction of the microfluidic chip.

$$\dot{Q}_{source} = \dot{Q}_{down} \frac{R_{down} + R_{up}}{R_{down}}$$

$$\dot{Q}_{down} = \dot{Q}_{TEG} \frac{R_{PDMS2} + R_{TEG}}{R_{PDMS2}}$$

The expression for the heat transfer fraction of the microfluidic channel:

$$\frac{\dot{Q}_{TEG}}{\dot{Q}_{source}} = \frac{R_{down}}{R_{down} + R_{up}} \frac{R_{PDMS2}}{R_{PDMS2} + R_{TEG}}$$

The resistances were expressed as thermal resistances $R_{conduction} = l / k_{material} A_{surface}$ and $R_{convection} = 1 / h_{convection} A_{surface}$. And the values used for the calculations are represented in the table below:

Table S6 Values used in the lumped element model.

Material	k [W/mK]	Density [kg/m^3]	Specific heat [J/kgK]
PDMS	0.16	965	1460
TEG (Bi2Te3)	1.2	7700	154
PMMA	0.2	1185	1466
Glass	1.38	2200	770
h_{water}	9008.4 W/m^2K		

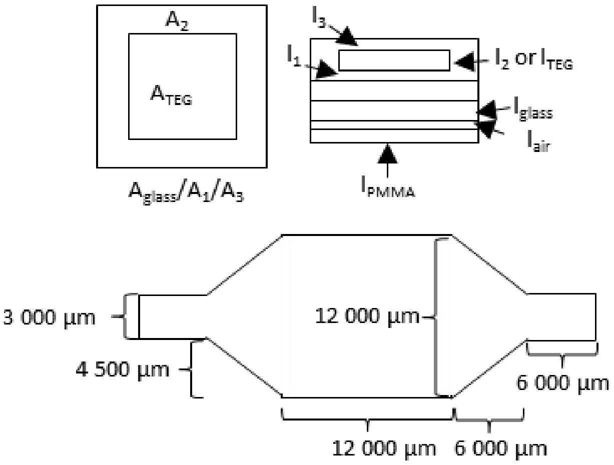

Figure S9 *Schematic (top view and side view) of the values used in the lumped element model. Dimensions of the microfluidic channels both sensing and control (320 µm height).*

Table S7 Geometric values used in the lumped element model

Area· 10^{-4} [m^2]		Length · 10^{-6} [m]	
A_1	1.44	l_1	150
A_2	0.44	l_2	500
A_3	1.44	l_3	150
A_{glass}	1.44	l_{glass}	170
A_{TEG}	1.00	l_{TEG}	500
A_{PMMA}	1.44	l_{PMMA}	1000
A_{air}	1.44	l_{air}	100

Time constants of the individual materials using $R_{conduction}=l/k_{material}A_{surface}$ for the thermal resistance, $C=A_{surface}\,l\,\rho_{material}\,c_{material}$for the thermal capacitance, and $\tau_{material\,layer}=RC$.

Table S8 Geometric values to approximate the individual time constants

Material layer	Thermal resistance	Thermal capacitance	Time constant [s]
PDMS layer 1	6.5	$3.04 \cdot 10^{-2}$	0.20
TEG	4.2	$5.93 \cdot 10^{-2}$	0.25
PDMS layer 2	71	$3.10 \cdot 10^{-2}$	2.20
PDMS layer 3	6.5	$3.04 \cdot 10^{-2}$	0.20
Glass	0.9	$4.55 \cdot 10^{-2}$	0.04
PMMA	34.7	$2.50 \cdot 10^{-1}$	8.68

8. Hydrodynamic and Thermal Entry Lengths

The hydrodynamic and thermal entry length were determined for all the different flow rates of our experiments.

The Reynolds number:

$$\Re ¿ \frac{\rho uL}{\mu}=\frac{\rho FL}{\mu A}$$

Re is the Reynolds number, which helps predict whether or not the flow is in the laminar or turbulent regime. $\Re \geq 2000$ is considered turbulent. With ρ as the density, μ as the dynamic viscosity, F as the flow rate, L as the characteristic length, and A the surface of the channel.

The Prandtl number:

$$Pr ¿ \frac{c_p \mu}{k}$$

Pr is the Prandtl number which is the ratio between momentum diffusivity and thermal diffusivity. With c_p as the specific heat, μ as the dynamic viscosity, and k as the thermal conductivity. In our case, the Prandtl number was determined to be 4.66 at $37^o C$.

The hydrodynamic entry length [6]:

$$L_h = 0.05 \Re L$$

The thermal entry length [6]:

$$L_{th} = 0.05 \Re \, Pr \, L = Pr \, L_h$$

In all cases the characteristic length $L \gg L_{th}, L_h$, as the characteristic length in our system was determined as $L = \sqrt{hb} = \sqrt{A} = 0.002\,m$ with$w = 12000\,\mu m$ and $h = 320\,\mu m$.

Table S9 Table of the calculated $\Re$***,*** L_{th}***, and*** L_h

Flow rate [$\frac{m^3}{s}$]	Reynolds number	Thermal entry length [μm]	Hydrodynamic entry length [μm]
$1.875 \cdot 10^{-10}$	0.044	6.4	1.4
$4.271 \cdot 10^{-10}$	0.100	14.5	3.1
$1.125 \cdot 10^{-9}$	0.263	38.3	8.2
$1.646 \cdot 10^{-9}$	0.385	56.0	12.0

9. Convective Heat Transfer Coefficient

The heat transfer coefficient used in the lumped element model (LEM) was calculated analytically according to Muzychka et al.[7]. Our microfluidic chip is in the fully developed flow region with the characteristic length larger than both the hydrodynamic entry length and the thermal entry length $L \gg L_{th}, L_h$. According to Yovanovic and Muzychka [8], the friction factor Reynolds number used to determine the Nusselt number is only dependent on the aspect ratio $\varepsilon = 0.0267$ of the channel dimensions:

$$f\,\Re_{\sqrt{A}} ¿ \frac{12}{\sqrt{\varepsilon}\,(1+\varepsilon) ¿¿}$$

The Nusselt number was then determined as:

$$N\,u_{\sqrt{A}} = C_1\left(\frac{f\,\Re_{\sqrt{A}}}{8\sqrt{\pi}\,\varepsilon^{\gamma}}\right) = 28.5$$

Where γ is a shape parameter with a value of $\frac{1}{10}$ as we have right angled corners in our microfluidic channel, and C_1 is a constant expressing that we assume uniform wall flux in our system in contrast to uniform wall temperature.

Through the relationship between the Nusselt number and the convective heat transfer coefficient we can express the convective heat transfer coefficient:

$$h = \frac{Nu_{\sqrt{A}} k}{L} = 9008.4 \frac{W}{m^2} K$$

We assume that the thermal conductivity of the liquid is the same as for water at $37^o C$, $k = 0.62 \frac{W}{mK}$, and the characteristic length $L = \sqrt{hb} = \sqrt{A} = 0.002\, m$.

10. Data Analysis of Raw Heat Flux Data

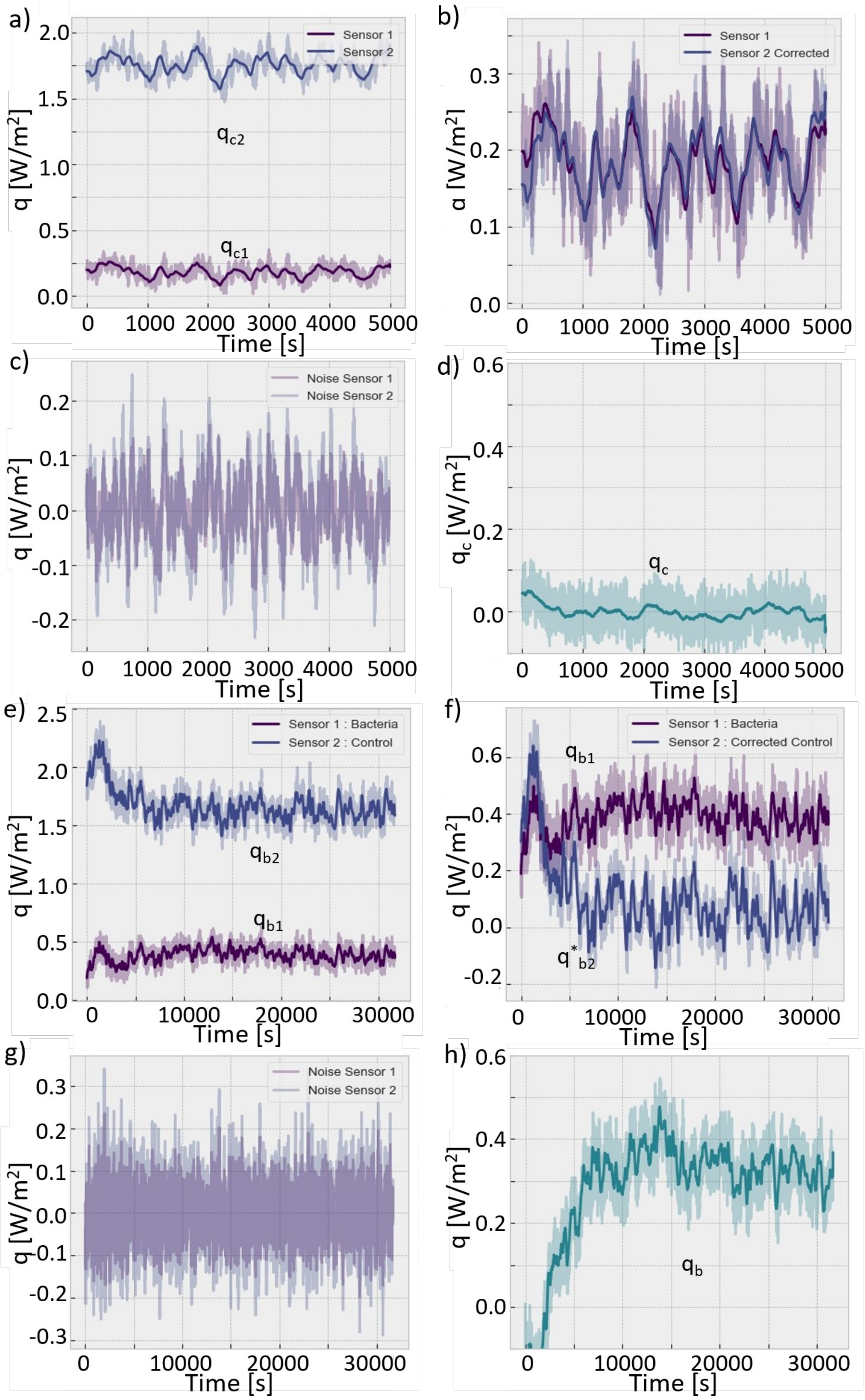

Figure S10 Extensive data analysis of heat flux experiments and differential compensation of bacterial thermal activity: a, b, c, d) *Calibration phase where both sensors contain LB*

medium only and ***e, f, g, h)*** *bacterial experiment with Sensor 1 containing bacteria and Sensor 2 containing LB media as compensation. In all figures, the raw data is shown in the lighter color, and a 200 point moving-average is shown in the darker color.* ***a, e)*** *Both show uncorrected raw data,* ***b)*** *shows the applied correction on one of the sensors according the compensation method* $q_{c2}^{¿} = L_c \cdot (q_{c2} - \bar{q}_{c2}) + \bar{q}_{c1}$*, and* ***f)*** *shows the applied correction according to* $q_{b2}^{¿} = L_c \cdot (q_{b2} - \bar{q}_{b2}) + \bar{q}_{c1}$*.* ***c, g)*** *Both show the left over noise upon correction.* ***d, h)*** *show the differentially compensated heat flux in the calibration experiment* $q_c = q_{c1} - q_{c2}^{¿}$ *and bacterial experiment* $q_b = q_{b1} - q_{b2}^{¿}$ *phase respectively.*

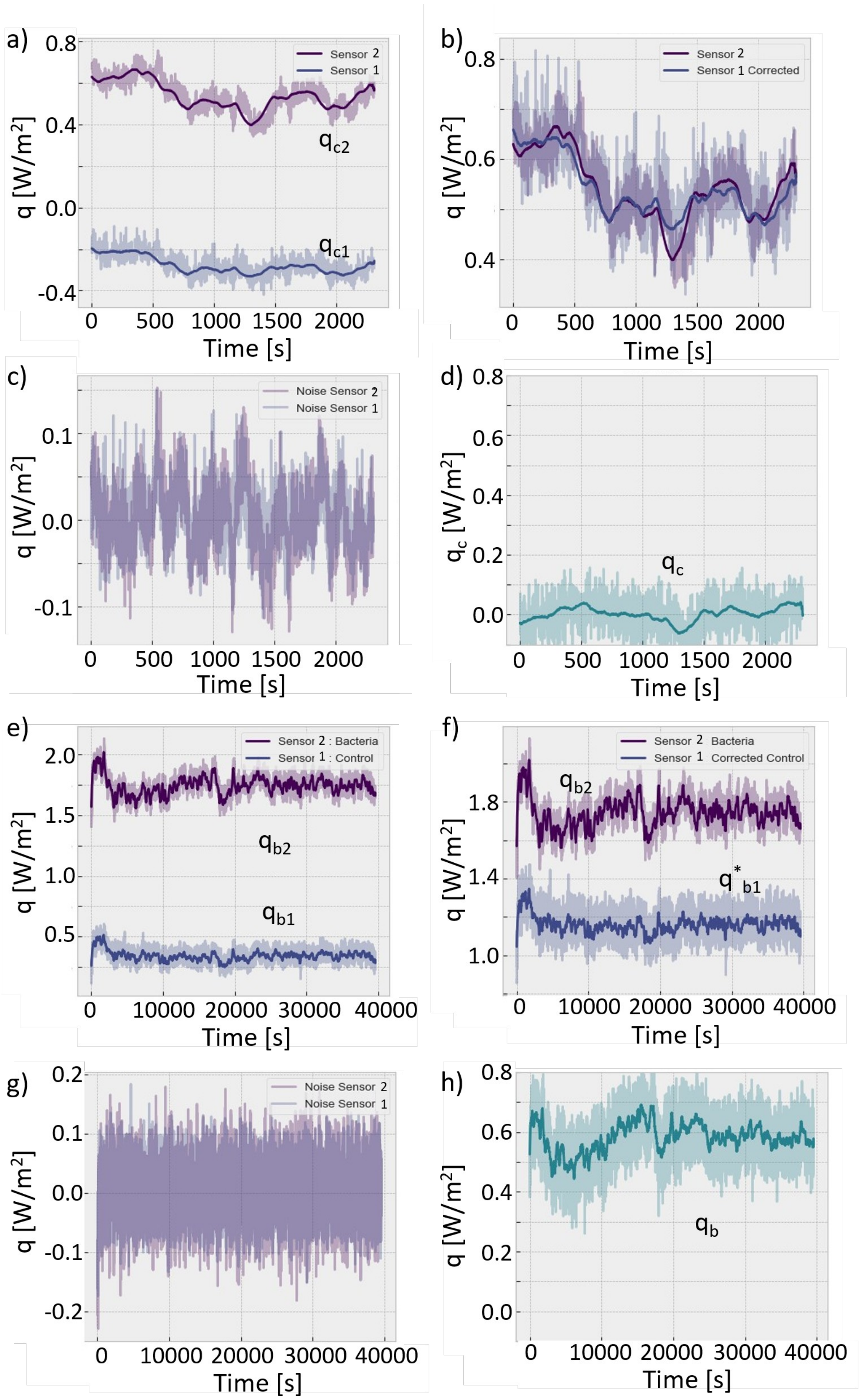

Figure S11 Extensive data analysis of heat flux experiments and differential compensation of bacterial thermal activity: *Second experiment after sterilization.* ***a, b, c, d)*** *Calibration phase where both sensors contain LB medium only and* ***e, f, g, h)*** *bacterial experiment with Sensor 1*

only containing LB as compensation and Sensor 2 containing bacteria. In all figures, the raw data is shown in the lighter color, and a 200 point moving-average is shown in the darker color. ***a, e)*** *Both show uncorrected raw data,* ***b)*** *shows the applied correction on one of the sensors according the compensation method* $q^{¿}_{c1} = L_c \cdot (q_{c1} - \bar{q}_{c1}) + \bar{q}_{c2}$*, and* ***f)*** *shows the applied correction according to* $q^{¿}_{b1} = L_c \cdot (q_{b1} - \bar{q}_{b1}) + \bar{q}_{c2}$*.* ***c, g)*** *Both show the left over noise upon correction.* ***d, h)*** *show the differentially compensated heat flux in the calibration experiment* $q_c = q_{c2} - q^{¿}_{c1}$ *and bacterial experiment* $q_b = q_{b2} - q^{¿}_{b1}$ *phase respectively. As can be seen, a thermal event upon the addition of bacteria occurred, acting out differently in the two sensors inducing a slight fluctuation in the beginning of the dataset.*

The equations used to correct for the background fluctuations is shown below.

The offset between the two sensors (sensor 1 and sensor 2) in the calibration phase (Fig. S11 a). It is determined by taking the average over 200 data points (sampling frequency ≈ 0.7 Hz):

$$q_{c2-c1} = \bar{q}_{c2} - \bar{q}_{c1}$$

Looking at both Fig. S11 a) and Fig. S12 a), each from different experiments (Fig. S4 e): Sensor 1 – bacteria, Sensor 2 – control; Fig. S12 e): Sensor 1 – control, Sensor 2 – bacteria) one can clearly identify that there is a correlation in the background fluctuations of both sensors, however, the background fluctuations influence the sensors to a different degree. Thereby, we can define a background fluctuation ratio:

$$L_c = \sigma_{c1} / \sigma_{c2}$$

With these two values, the offset q_{c2-c1} and the ratio L_c, we can define a corrected value for one of the sensors during the calibration, which are plotted in Fig. S11 b) and Fig. S12 b), respectively for the two different experiments:

$$q^{¿}_{2c} = (q_{2c} - \bar{q}_{2c}) L_c + \bar{q}_{c2} - (\bar{q}_{c2} - \bar{q}_{c1}) = (q_{2c} - \bar{q}_{2c}) L_c + \bar{q}_{c1}$$

The then calculated difference in heat flux between the two sensors can be expressed as, and are plotted in Fig. S11 d) and Fig. S12 d):

$$q_c = q_{c1} - q_{c2}^{¿}$$

The same correction was done upon addition of bacteria yielding Fig. S11 h) and Fig. S12 h).

$$q_{b2}^{¿} = \left(q_{b2} - \bar{q}_{b2}\right) L_c + \bar{q}_{c1}$$

The heat flux left over after the background fluctuations were cancelled out represents the heat produced by the bacterial growth.

$$q_b = q_{b1} - q_{b2}^{¿}$$

In the case of Fig. S12, the signal of sensor 1 (control in this case) was corrected. This means q_{c1} was corrected to $q_{c1}^{¿}$ and q_{b1} was corrected to $q_{b1}^{¿}$, by the exact same equations as shown above.

The heat measured is the heat flux measured times the surface area, A_{sensor}, of the sensor (10 mm x 10 mm):

$$\dot{Q}_b = q_b \, A_{sensor}$$

11. Overall Calculation of Single Bacterium Thermal Power

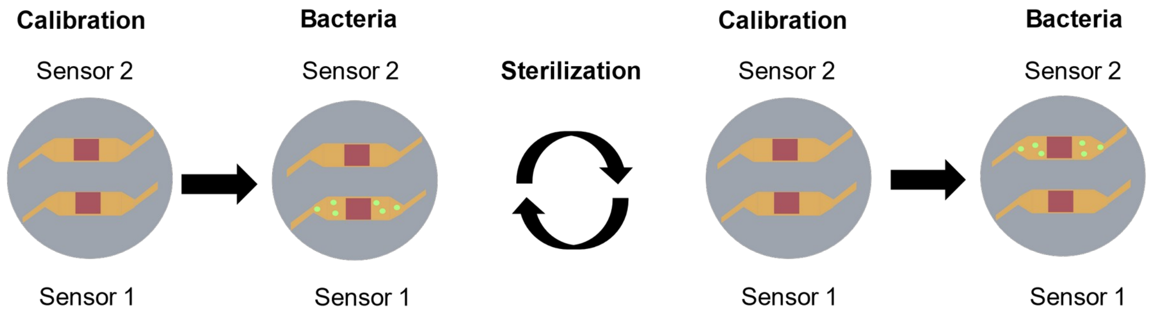

Figure S12 Schematic for the determination of the single bacterium thermal power: *The left side showing the configuration of the data shown in Fig. S11, and the right side representing the configuration of the data shown in Fig. S12.*

The combination of the bacterial experiment and the methyl paraben experiments to be able to extract the thermal power of a single bacterium of the system.

The exponential region of the bacterial growth is determined by the optical density measurement and it is fitted by an exponential function as shown in Fig. S2:

$$N_t = N_0 e^{\mu_{OD} t}$$

$$\ln(N ¿¿ t) = \ln(N ¿¿ 0) + \mu_{OD} t ¿¿$$

The error of the fit is calculated by subtracting the data from the fit and taking a standard deviation of the remaining noise as shown in section 6 in the SI (Error propagation for the single bacterium thermal power).

In the same time region, the heat flux measured is fitted to an exponential function:

$$\dot{Q} = \dot{Q}_0 e^{\mu_Q t}$$

$$\ln(\dot{Q}) = \ln(\dot{Q} ¿¿ 0) + \mu_Q t ¿$$

The error of the fit was calculated by subtracting the averaged data over 200 data-points from the fit and taking a standard deviation of the remaining noise as shown in section 6 the SI (Error propagation for the single bacterium thermal power). Where $\dot{Q} = ¿\, \dot{Q}_b [t_{\exp start} : t_{\exp end}]$, representing the time region where the exponential growth is identified from the optical density measurement. The single bacterium thermal power was calculated as:

$$\dot{Q}_{bacteria} = \frac{\dot{Q} / \chi_{mp}}{N_t OD_{ratio} V_{channel}}$$

Where $\dot{Q}$ is the differentially compensated thermal power measured in the exponential region of bacterial growth, χ_{mp} is the heat transfer fraction, N_t as the optical density in the same exponential time frame as $\dot{Q}$, OD_{ratio} is the measured optical density conversion to bacteria (as shown in Fig. S8), and $V_{channel}$ is the volume of the channel.

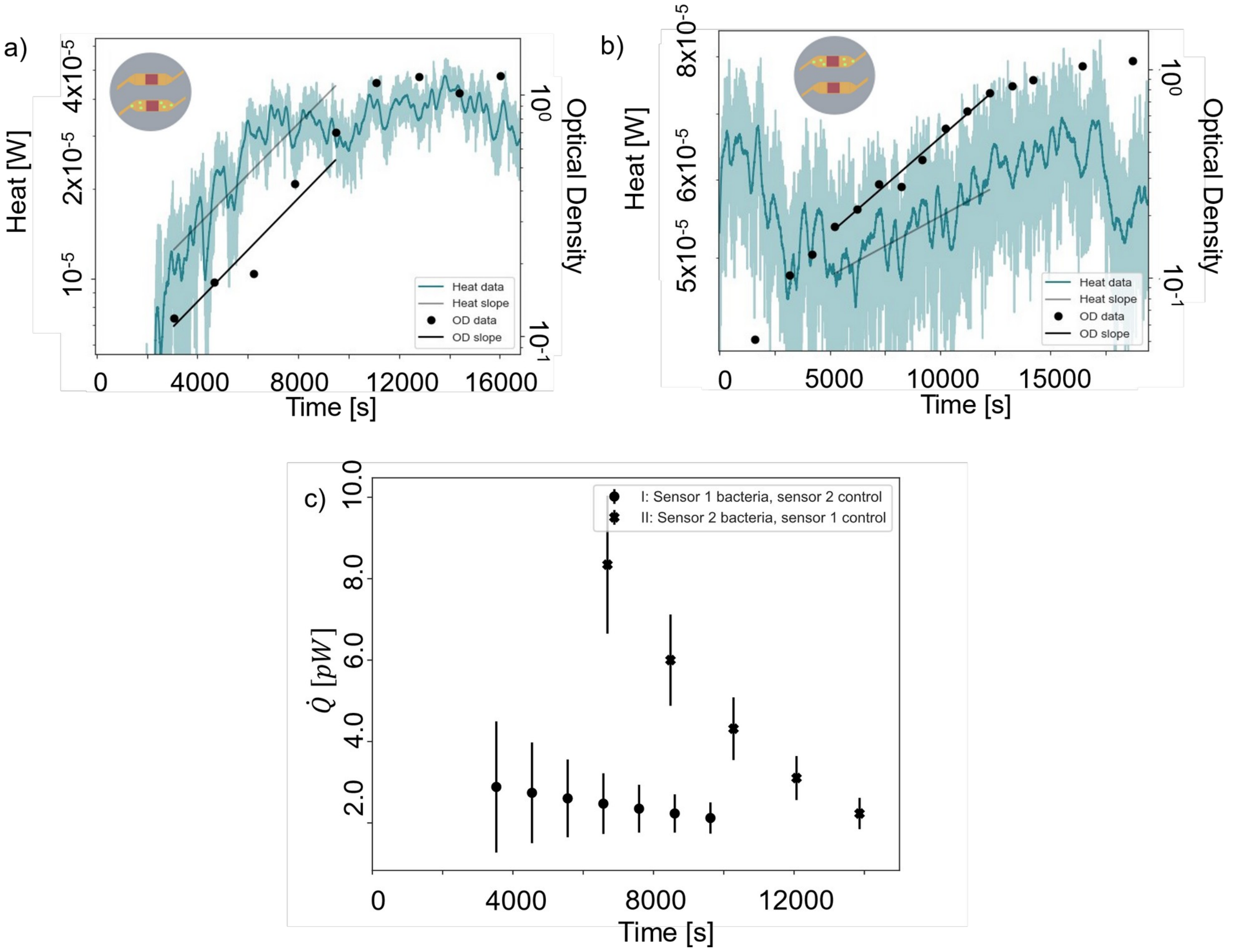

***Figure S13 Thermal power measured in the exponential bacterial growth region**: **a, b)** Exponential bacterial growth region indicated by exponential fit for both optical density data and differentially compensated thermal power. **a)** Shows the same data as shown in Fig. 3 d), Fig. 4 a), and Fig. S4 h), and **b)** showing the data after sterilization and switching of the functions of Sensor 1 and Sensor 2 as to compensation and bacterial experiment as shown and elaborated on in Fig. S11 h). **c)** Shows the extracted singular bacterium thermal power in both experiments with respective error bars as calculated as discussed in section 6 in the SI.*

Both systems show decrease in singular bacterial thermal power upon increase in time. The error in the second experiment (II) is higher than that for the first experiment (I) due to the thermal fluctuation at the beginning of the second experiment.

***Table S10 Extension of Table 1 in the main text**: Values extracted from the repeated experiment after sterilization and reversing the function of the sensing and control sensor. The rate increase in thermal power in both the experiments varied due to the different thermal response to an external change, as already mentioned in the main text.*

**System**	$\mu_{OD}[s^{-1}]$	$\mu_{\dot{Q}}[s^{-1}]$	$\chi_{mp}[\%]$	$\dot{Q}_{bacterium}[pW]$
Sensor 1: Bacteria	$2.81 \cdot 10^{-4}$	$1.97 \cdot 10^{-4}$	77 ± 8	1.3 – 4.5
Sensor 2: Bacteria	$2.18 \cdot 10^{-4}$	$2.79 \cdot 10^{-5}$	52 ± 9	1.9 – 4.0

12. OD to bacteria/mL Calibration

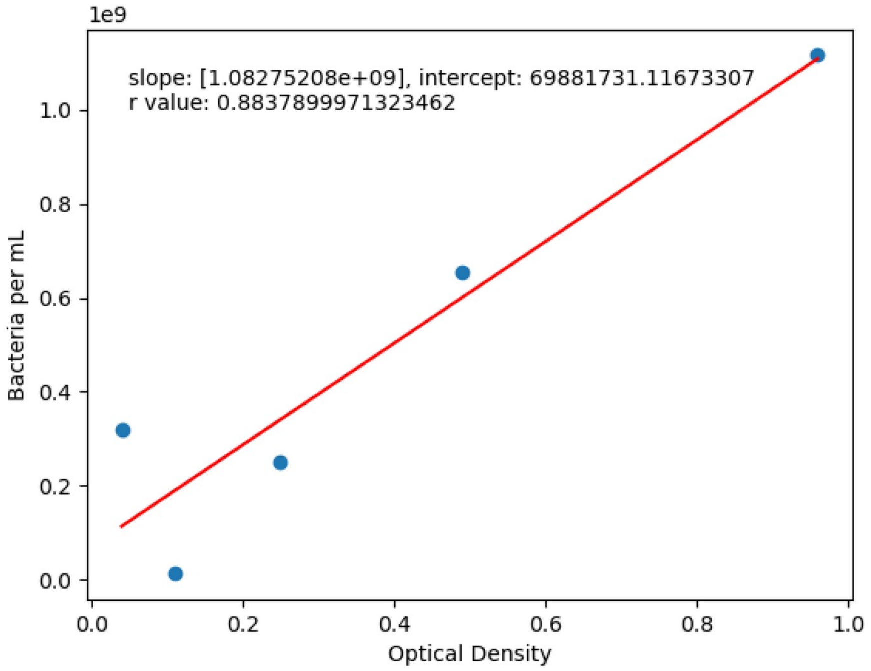

Figure S14 Determination of OD conversion factor: *Experimental determination of bacteria per mL by plating and optical density measurement.*

Plates were prepared using LB agar and samples were taken at 30 min intervals and the OD was measured. 10-fold dilutions were made with PBS 7 times and plated to be counted using ImageJ.

To determine the bacteria concentration corresponding to the measured optical density. Plates at different concentrations were made at different measured optical densities. The function was used to determine the amount of bacteria measured using optical density. The slope found by linear regression was $OD_{ratio} = 1.08 \cdot 10^9$ which fits the standard value used to convert OD to bacteria/mL.

13. Sensitivity, Resolution, and Limit of Detection

The standard deviation (SD) of the raw data in relation to the average over 200 data points, with sampling frequency $\approx 0.7\,Hz$, was determined as $0.077\frac{W}{m^2}$. The standard deviation of the averaged data was $0.015\frac{W}{m^2}$.

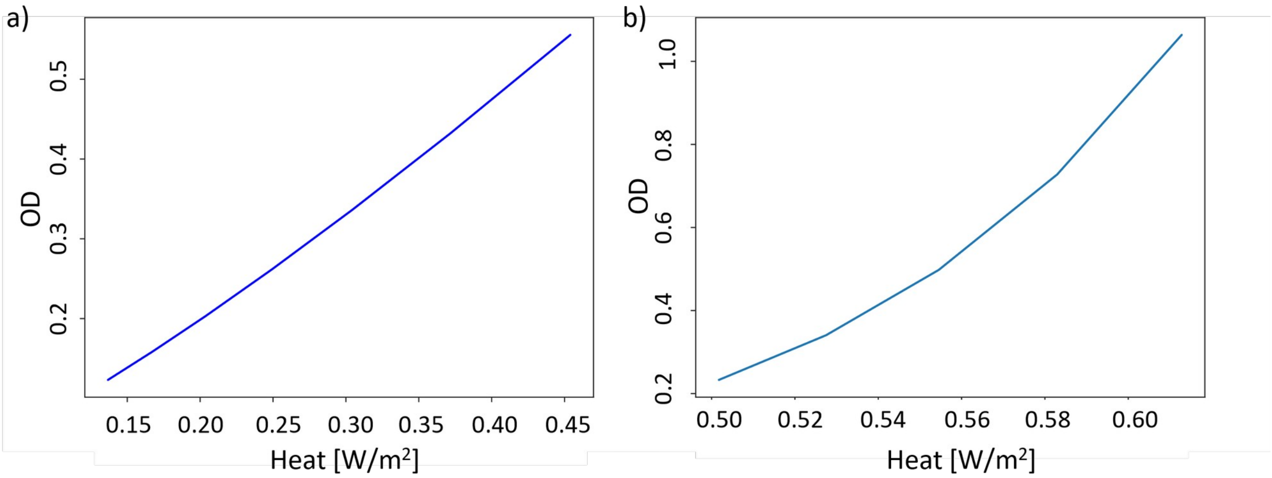

Figure S15 Sensitivity of both experimental configurations: *a) Heat flux vs OD in the exponential region for bacteria underneath sensor 1. b) Heat flux vs OD in the exponential region for bacteria underneath sensor 2.*

The slope of the trend line between heat and OD is the sensitivity. As previously mentioned, the second experiment (shown in Fig. S11 h) and S13 b)) was affected by a thermal event upon the addition of bacteria, thereby causing a change in the sensitivity of the system and thus also changing the limit of detection. In the case of a nonlinear trend line, the sensitivity is the derivative of the fit of the trend line. In our data, the sensitivity of the first experiment is determined as $\frac{\partial N_t}{\delta \dot{Q}_e}=1.36\frac{OD/W}{m^2}$ with the trend line is close to linear, for the second experiment is determined

as $\frac{\partial N_t}{\delta \dot{Q}_e} = 7.42 \frac{OD/W}{m^2}$. Converted to $\frac{OD_{ratio} \partial N_t}{\partial \dot{q}_e} = 1.47 \times 10^{13} \frac{¿bacteria}{W}$, respectively. The limit of detection is defined as the standard deviation divided by the sensitivity.

***Table S11** The different values for standard deviation, sensitivity, and limit of detection in both our experiments. Standard deviation taken*

Experiment	Standard Deviation	Sensitivity	Limit of Detection
Sensor 1	$0.015 \frac{W}{m^2}$	$0.73 \frac{OD/W}{m^2}$	$0.021\,OD$
	$1.52 \cdot 10^{-6}\,W$	$6.79 \times 10^{-14} \frac{W}{¿bacteria}$	$22435882\,¿bacteria$
Sensor 2	$0.022 \frac{W}{m^2}$	$0.13 \frac{OD/W}{m^2}$	$0.162\,OD$
	$2.12 \cdot 10^{-6}\,W$	$1.25 \times 10^{-14} \frac{W}{¿bacteria}$	$174431495\,¿bacteria$

The sensitivity of the second experiment was lower due to an external thermal effect of the system.

14. Thermal Power of Biofilms

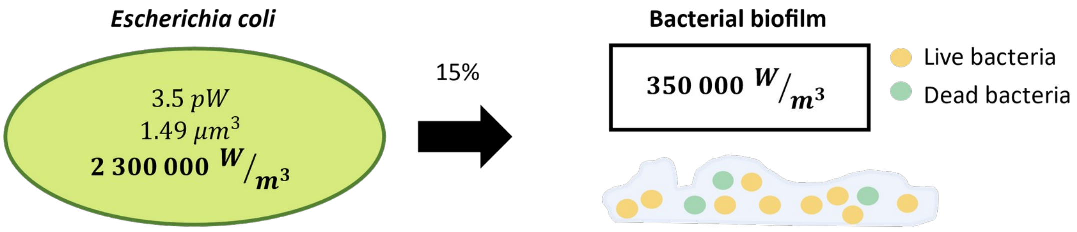

Figure S16 Sketch of determination of thermal density of a bacterial biofilm: *Heat density of single bacterium and bacterial biofilm.*

One *E. coli* bacteria has been shown to produce 3.5 pW in literature[1] (7.8 pW [2] has been found in literature for LBG media), and to have a volume of 1.49 µm^3 in literature[3]. Thereby, the heat density of an *E. coli* is around 2 300 000 W/m^3. The amount of active bacteria in a biofilm has been shown to be 15 % of a biofilm[4] corresponding to 350 000 W/m^3.

15. Comparison Between the Bacterial Growth in the Source in Comparison to After Travelling Through the Tubing and the Chip

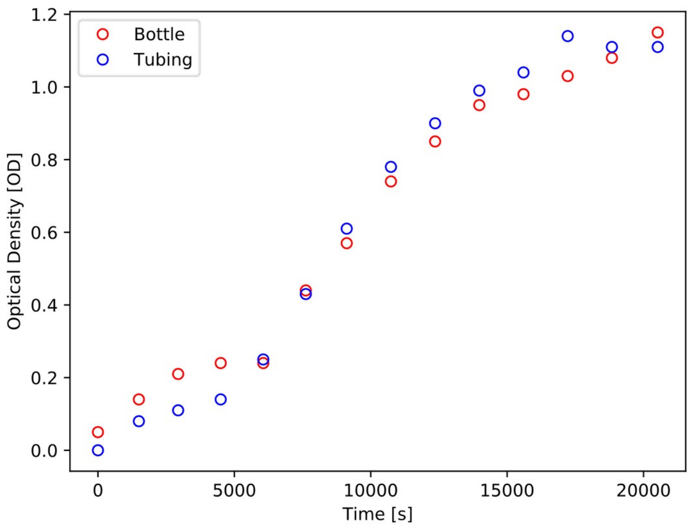

Figure S17 Comparison between OD measurement before and after travelling through tubing and microfluidic chip: *Optical density measurement comparison between the number of bacteria in the aerated bottle, and at the end of the tubing corrected for the travel time.*

The optical density was measured in parallel between the growth within the bottle and at the end of the tubing. This was to determine any potential discrepancies in the optical density measurement assumed in the channel and measured by collecting samples at the end of travelling through the tubing and the chip. A liquid sample was taken every 25 minutes and placed in an ice container to halt the bacterial growth until the optical density was measured. 100 cm of PTFE tubing was used in addition to the peristaltic pump tubing of 30 cm. The flow rate was $25.6\,\frac{\mu L}{min}$. We corrected for the travel time within the tubing (with known inner diameters). We observed a slight difference in

the bacterial population during the initial lag phase (≈ 6000 s), and during the saturation phase in the end. During the exponential growth phase there seemed to be good overlap between the values within the bottle and the tubing. The initial discrepancy during the lag phase could be due to the bacteria initially sticking to the inner wall of the tubing, and the latter could be due to the release of the bacteria that initially got stuck during the lag phase. This effect could also have an influence on the decreasing heat per single bacterium as shown in Fig. S13 c). As time passes, if more bacteria are released from sticking to the inner wall of the tubing, the heat per bacteria would decrease.

References

1. Higuera-Guisset, J. *et al.* Calorimetry of microbial growth using a thermopile based microreactor. *Thermochim. Acta* **427**, 187–191 (2005).
2. Guosheng, L., Yi, L., Xiangdong, C., Peng, L. & Ping, S. Study on interaction between T4 phage and Escherichia coli B by microcalorimetric method. **112**, 137–143 (2003).
3. Kubitschek, H. E. Growth During the Bacterial Cell Cycle: Analysis of Cell Size Distribution. *Biophys. J.* **9**, 792–809 (1969).
4. Philipps, R., Kondev, J., Theriot, J. & Garcia, H. G. *Physical Biology of the Cell*.
5. O'Neill, M. A. A. *et al.* The base catalysed hydrolysis of methyl paraben: A test reaction for flow microcalorimeters used for determination of both kinetic and thermodynamic parameters. *Thermochim. Acta* **399**, 63–71 (2003).
6. Cengel, Y. A. *Heat Transference a Practical Approach (Second Edition)*. *McGraw-Hill* **4**, (2004).
7. Muzychka, Y. S. & Yovanovich, M. M. Laminar forced convection heat transfer in the combined entry region of non-circular ducts. *J. Heat Transfer* **126**, 54–61 (2004).
8. Yovanovich, M. M. & Muzychka, Y. S. Solutions of poisson equation within singly and doubly connected prismatic domains. *Natl. Heat Transf. Conf. 1997* (1997). doi:10.2514/6.1997-3880
